# Analysis and Design of Sensor-Driver-Aware Integral Nonsingular Terminal Sliding Mode Control for Buck Converter Power Interfaces in Actuator Systems

**DOI:** 10.3390/mi17070829

**Published:** 2026-07-11

**Authors:** Weiqi Zhang, Fan Ping, Yingbo Han, Kai Song, Chuanyu Sun

**Affiliations:** 1 School of Electrical Engineering and Automation, Harbin Institute of Technology, Harbin 150001, China; 23b906013@stu.hit.edu.cn; 2 NARI Technology Co., Ltd., Nanjing 211106, China; pingfan@sgepri.sgcc.com.cn; 3 Faculty of Computing, Harbin Institute of Technology, Harbin 150001, China; 2024112677@stu.hit.edu.cn

**Keywords:** buck converter, micro-actuator power interface, sliding mode control, sensor-driver dynamics, multi-source disturbances, phase trajectory, response-time estimation

## Abstract

Buck converter power interfaces are commonly used as local voltage regulation units in compact actuator-driven microsystems, where the regulated voltage needs to remain stable under input, load, and circuit-level disturbances. In practical control loops, passive-parameter variations, external perturbations, and non-ideal sensor-driver dynamics may distort feedback signals, delay effective duty-cycle action, and degrade the transient response of conventional robust controllers. Motivated by this issue, this paper presents the analysis and design of a sensor-driver-aware integral nonsingular terminal sliding mode control (INTSMC) method for a buck converter power interface under multi-source disturbances. A control-oriented averaged model is first constructed by incorporating converter parameter perturbations, load-side disturbances, Hall sensor dynamics, and isolated driver characteristics into a unified representation. Based on this model, an integral nonsingular terminal sliding surface is designed to improve voltage tracking performance while avoiding singularity in the reaching process. The corresponding control law is further arranged in a pulse-width modulation-realizable duty-cycle form, making it suitable for digital converter control. In addition, a phase-trajectory-based response-time estimation method is introduced to analyze the influence of initial states, disturbance levels, and hardware dynamic parameters on the closed-loop reaching behavior. Simulation studies under different operating conditions are carried out to evaluate the proposed controller. Simulation and experimental results show that the proposed method achieves a settling time within 33 ms, a steady-state voltage error within 0.01 V, and a measured efficiency of 83.5%~88.9%, indicating its feasibility for robust power regulation in micro-actuator-oriented microsystems where sensor-driver dynamics cannot be ignored.

## 1. Introduction

Compact actuator-driven microsystems, such as micro-actuation modules, micro-positioning stages, micro-pumps, and miniature mechatronic platforms, often rely on local power regulation units to provide stable and responsive voltage support for sensing, driving, and control circuits. In such systems, the buck converter is a practical choice because of its simple structure, high conversion efficiency, and compatibility with digital pulse-width modulation (PWM) implementation [1,2]. Unlike large-scale power conversion applications, microsystem-oriented converters usually operate under tighter space, power, and dynamic-response constraints [3]. Variations in the input source, load demand, and circuit parameters may therefore have a direct influence on the quality of the regulated voltage, especially when the converter is used to support actuator-related modules requiring fast and reliable electrical response [4,5].

For this type of converter, the control loop is seldom affected by a single uncertainty source [6]. Input-voltage fluctuations and load changes alter the operating point of the power stage, while the aging or tolerance of passive components changes the local dynamic characteristics of the circuit [7]. In addition, the measured current and voltage used for feedback are usually obtained through sensor circuits with their own dynamic response, and the duty-cycle command must pass through a gate-driving stage before it acts on the switch [8]. These non-ideal links may introduce signal distortion or action delay, making the actual closed-loop behavior different from that predicted by an ideal averaged buck converter model [9].

Several control strategies have been reported for improving the dynamic performance of Buck and other DC-DC converters. Proportional–integral (PI) and proportional–integral–derivative (PID) controllers are commonly adopted because of their simple structure and low implementation cost, and PI/PID schemes remain attractive in many converter applications where the operating range is relatively narrow [10]. However, their performance may deteriorate when the operating point, load condition, or circuit parameters change significantly. Model predictive control (MPC) provides a more flexible framework for handling switching constraints and transient optimization, and MPC-based converter control can achieve good dynamic performance when an accurate model and sufficient computational resources are available [11]. Active disturbance rejection control (ADRC) and other observer-based methods can estimate lumped disturbances and compensate for them online, but the performance of ADRC is closely related to the selection of observer bandwidth and controller parameters, especially in noisy measurement environments [12]. In comparison, sliding mode control (SMC) has been widely investigated for power converters because of its inherent robustness against matched uncertainties and load variations. SMC-based methods are therefore suitable for converter systems subject to input fluctuation and load disturbance [13]. Nevertheless, conventional SMC may introduce chattering and usually involves a compromise between fast reaching, steady-state accuracy, and smooth duty-cycle generation. To improve the convergence behavior, terminal sliding mode control (TSMC) and nonsingular terminal sliding mode control (NTSMC) have been introduced into converter control, and NTSMC can provide faster nonlinear convergence while avoiding the singularity problem in the sliding dynamics [14,15].

Recent studies have improved the robustness of DC-DC converter control from different perspectives. In [16], an integral sliding mode control method was designed for a DC-DC boost converter with uncertainties, where the integral sliding manifold and discontinuous compensation improve voltage regulation, but the design is mainly based on a linearized uncertainty-augmented model. Gong and Fei proposed an adaptive neural backstepping TSMC scheme for a buck converter, using a recurrent neural network to approximate system uncertainties and TSMC to achieve finite-time convergence [17]. Although good tracking performance is obtained, the neural compensation structure increases the controller complexity. Liu et al. combined nonsingular fast TSMC control with a fuzzy neural network and a disturbance observer to improve disturbance compensation and transient response in buck converter control [18]. However, the sensor and driver stages are still not explicitly included in the closed-loop model. In [19], a fixed-time extended-order TSMC method was developed for DC microgrids with constant-switching-frequency PWM implementation and adaptive feedforward tuning, but its main focus is microgrid-level converter regulation rather than hardware-link-induced feedback and actuation deviations. Wei and Zhang proposed a high-order fully actuated robust controller for buck converters feeding constant power loads, where an uncertainty boundary estimator and a differential state observer enhance large-signal robustness [20]. This method effectively addresses load-induced instability, but it does not focus on sensor-driver dynamic effects in compact control loops. In addition, topology-oriented research on bidirectional boost/buck converters has shown that switched-capacitor structures and complementary PWM modulation can improve voltage gain, reduce device stress, and support stable operation during load transitions and power-flow reversal [21]. These studies provide useful references for robust converter control and implementation, yet the joint treatment of multi-source disturbances, sensor-driver dynamics, PWM-realizable integral nonsingular terminal sliding mode control (INTSMC), and phase-trajectory (PT)-based response-time analysis remains insufficient.

Although the above methods have achieved notable progress, several issues still need further study. Most sliding-mode-based converter controllers are developed from the ideal or simplified power-stage model, while the dynamic effects of current/voltage sensors and gate-driving circuits are often neglected. Meanwhile, input fluctuations, load disturbances, and parameter variations are usually treated as lumped uncertainties, so their coupled influence with non-ideal sensing and driving dynamics on the closed-loop transient process is not fully revealed. Moreover, existing performance evaluations mainly rely on time-domain waveforms and numerical indices, with less attention paid to the state-PT evolution during the reaching process. Therefore, a control-oriented framework that combines multi-source disturbance modeling, sensor-driver-aware robust control, PWM-realizable implementation, and PT-based response-time characterization is still needed for buck converter power interfaces in compact actuator-driven microsystems.

Motivated by the above discussion, this paper presents the analysis and design of a sensor-driver-aware INTSMC method for a buck converter power interface used in compact actuator-driven microsystems. Different from studies that mainly focus on ideal power-stage modeling or lumped disturbance compensation, this work considers the influence of sensing and driving dynamics on the closed-loop response. The main contributions are summarized as follows:

1. A sensor-driver-aware buck converter power-interface model for micro-actuator-driven microsystems is established by incorporating input-voltage fluctuation, load disturbance, circuit parameter variation, current/voltage sensor dynamics, and gate-driving dynamics into a unified control-oriented framework.
2. An INTSMC method is designed for voltage regulation under multi-source disturbances. The control law is expressed in a PWM-realizable duty-cycle form for digital converter control.
3. A PT-based response-time analysis method is introduced to characterize the reaching process under different initial states, disturbance levels, and sensor-driver dynamic parameters.
4. Comparative simulation studies are conducted under different operating conditions to evaluate the proposed method in terms of transient response, steady-state deviation, and disturbance rejection capability.

The remainder of this paper is organized as follows. Section 2 presents the sensor-driver-aware buck converter model with multi-source disturbances. Section 3 gives the design of the proposed INTSMC controller and the corresponding stability analysis. Section 4 introduces the PT-based response-time estimation method. Section 5 provides comparative simulation results under different operating conditions. Finally, Section 6 concludes this paper.

## 2. Modeling of the Buck Converter with Multi-Source Disturbances and Sensor-Driver Dynamics

### 2.1. Averaged Model of the Buck Converter

**Figure 1:**
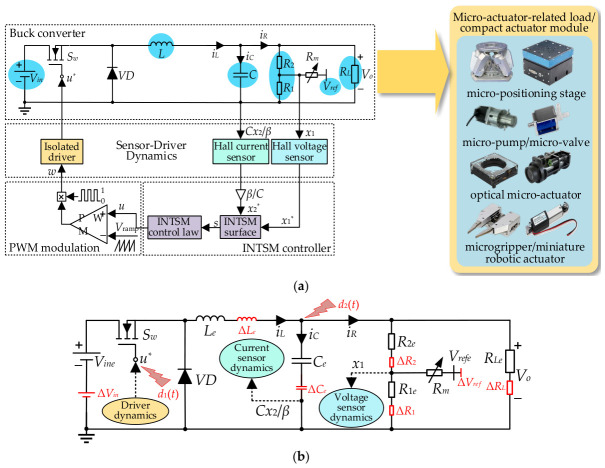
Sensor-driver-aware buck converter power interface for micro-actuator-oriented microsystems: (**a**) INTSMC-controlled buck converter with micro-actuator-related load; (**b**) equivalent circuit model with sensor-driver dynamics and multi-source disturbances.

Figure 1a shows the circuit topology of a general buck converter. It consists of a DC input voltage source *V_in_* with 0 ≤ *V_in_* ≤ *V_in_*_max_, where *V_in_*_max_ is the maximum of *V_in_*; a controlled power switch *S_W_*, which is usually implemented using a MOSFET and IGBT, with the PWM technology with the constant-frequency ramp single *V*_ramp_ adopted for *S_W_* to realize the duty ratio control, i.e., the duty ratio *u* ∈ [0, 1]; a diode *VD*; a filter inductor *L*; a filter capacitor *C*; a nominal load resistor *R_L_*; divider resistors *R*_1_, *R*_2_ with the ration *β* = *R*_1_/(*R*_1_ + *R*_2_); the variable resistor *R_m_* is used to provide the reference voltage difference *V_ref_*. The currents flowing through *L*, *C*, and *R_L_* (with *R*_1_ and *R*_2_) can be labeled as *i_L_*, *i_C_*, and *i_R_*, respectively, and *V_o_* is the output voltage of the buck system.

Further, the “*” terms are system variables that take sensor-driver dynamics into account in Figure 1a, such as u* and *w* (which is generated by PWM from *u*), which are the output and input control signals from the isolated driver, respectively. In the considered micro-actuator-oriented microsystem, the buck converter serves as a local power regulation interface for actuator-related driving and control circuits. The equivalent load *R_L_* is compact actuator modules and their peripheral circuits, where fast voltage recovery and limited current fluctuation are required for a reliable actuator-side electrical response.

For the controlled buck converter shown in Figure 1a, we assume that it operates in continuous conduction mode (CCM), meaning that the current flows through the *L*, denoted as *i_L_* ≠ 0. Based on Kirchhoff’s circuit law, we can express the model describing the ON/OFF operation of the buck converter as follows:

(1) $$\left\{ \begin{array}{l} \frac{di_{L}}{dt}=\frac{1}{L}\left(V_{in}-V_{o}\right)\\ \frac{dV_{o}}{dt}=\frac{1}{C}\left(i_{L}-\frac{V_{o}}{R_{L}}\right) \end{array}\right.$$

(2) $$\left\{ \begin{array}{l} \frac{di_{L}}{dt}=-\frac{1}{L}V_{o}\\ \frac{dV_{o}}{dt}=\frac{1}{C}\left(i_{L}-\frac{V_{o}}{R_{L}}\right) \end{array}\right.$$

We use u* = 1 and u* = 0 to accurately denote the switching conditions ON and OFF, respectively, for expressing the inherent switching characteristics of the buck converter shown in Figure 1a. By combining (1) and (2), we can deduce the equation that uniformly describes the averaging state-space model [22] of the buck converter as:

(3) $$\left\{ \begin{array}{l} \frac{di_{L}}{dt}=\frac{1}{L}\left(u^{*}V_{in}-V_{o}\right)\\ \frac{dV_{o}}{dt}=\frac{1}{C}\left(i_{L}-\frac{V_{o}}{R_{L}}\right) \end{array}\right.$$

We assume that *V_ref_* represents the reference output voltage of the buck system, which can be regarded as a constant voltage value, i.e., ${\dot{V}}_{ref}$ = 0. We define system state variables ***x*** = [*x*_1_, *x*_2_, *x*_3_]^T^, where *x*_1_ is the voltage error, *x*_2_ is the voltage error dynamics (or the rate of change of the voltage error), and *x*_3_ is the integral of voltage error (or the cumulative deviation of voltage error), which can be further expressed as:

(4) $$\{\begin{array}{l} x_{1}=\beta V_{o}-V_{ref}\\ x_{2}={\dot{x}}_{1}=\beta {\dot{V}}_{o}-{\dot{V}}_{ref}=\beta {\dot{V}}_{o}\\ x_{3}=\int_{0}^{t}x_{1}dt \end{array}$$

### 2.2. Buck Converter Model with Multi-Source Disturbances

In practical applications, multiple model uncertainties and external disturbances in the model of the buck converter (3) have to be considered, as shown in Figure 1b. For compact actuator-driven microsystems, these disturbances can be associated with supply fluctuation of the local power bus, actuator-related load variation, passive component tolerance in miniaturized circuits, and electromagnetic noise introduced by high-frequency switching and densely integrated signal paths.

Based on (3), the variations of *R_L_*, *V_in_*, *L*, *C*, and time-varying external disturbances *d*_1_(*t*) and *d*_2_(*t*) (e.g., measurement noise or electromagnetic interference [23]) are introduced as follows:

(5) $$\left\{ \begin{array}{l} \frac{di_{L}}{dt}=\frac{1}{L_{e}+\Delta L}\left[u^{*}\left(V_{ine}+\Delta V_{in}\right)-V_{o}\right]+d_{1}(t)\\ \frac{dV_{o}}{dt}=\frac{1}{C_{e}+\Delta C}\left(i_{L}-\frac{V_{o}}{R_{Le}+\Delta R_{L}}\right)+d_{2}(t) \end{array}\right.$$

where the terms with “Δ” are the corresponding disturbance variations of the nominal variables, the internal parameter disturbances Δ*R_L_*, Δ*L*, Δ*C* and external parameter disturbances Δ*V_in_* and Δ*V_ref_* are caused by parameter perturbations of the buck system and can be described as (and the Δ*V_ref_* in Figure 1 has also been considered for system integrity):

(6) $$\{\begin{array}{l} V_{in}=V_{ine}+\Delta V_{in},\;\left|\Delta V_{in}\right|\leq \psi_{Vin}\leq V_{ine};\;V_{ref}=V_{refe}+\Delta V_{ref},\;\left|\Delta V_{ref}\right|\leq \psi_{Vrefe}\leq V_{ref};\\ R_{L}=R_{Le}+\Delta R_{L},\;\left|\Delta R_{L}\right|\leq \psi_{RL}\leq R_{Le};\;L=L_{e}+\Delta L,\;\left|\Delta L\right|\leq \psi_{L}\leq L_{e};\\ R_{1}=R_{1e}+\Delta R_{1},\;\left|\Delta R_{1}\right|\leq \psi_{R1}\leq R_{1e};\;R_{2}=R_{2e}+\Delta R_{2},\;\left|\Delta R_{2}\right|\leq \psi_{R2}\leq R_{2e};\\ C=C_{e}+\Delta C,\;\left|\Delta C\right|\leq \psi_{C}\leq C;\\ \left|d_{1}(t)\right|\leq \psi_{d1},\;\left|{\dot{d}}_{1}(t)\right|\leq \psi_{\dot{d}1};\;\left|d_{2}(t)\right|\leq \psi_{d2},\;\left|{\dot{d}}_{2}(t)\right|\leq \psi_{\dot{d}2}; \end{array}$$

where *R_Le_*, *L_e_*, *C_e_*, *V_ine_*, *V_refe_*, *R*_1*e*_, and *R*_2*e*_ are the known estimates of the resistor, inductor, capacitor, input voltage, and reference voltage; ${\dot{d}}_{1}\left(t\right)$ and ${\dot{d}}_{2}\left(t\right)$ are derivatives of time-varying external disturbances *d*_1_(*t*) and *d*_2_(*t*); and *ψ_RL_*, *ψ_L_*, *ψ_C_*, *ψ_Vin_*, *ψ_Vref_*, *ψ_R_*_1_, *ψ_R_*_2_, $\psi_{{\dot{d}}_{1}}$ and $\psi_{{\dot{d}}_{2}}$ are constants. Since the parameters of the resistor, inductor, and capacitor cannot be guaranteed at the rated values in manufacturing, and aging or environmental temperature may also affect the parameters and the external input voltage signal of the buck system, uncertainties have to be considered.

Since passive parameter variations and source/load changes are usually slower than the switching dynamics and controller sampling process, their derivatives are assumed to be bounded and sufficiently small within one control interval [24]; therefore, the derivative of the corresponding perturbation terms can be approximated as zero, which provides convenience for system modeling, such as $\Delta {\dot{V}}_{ref}$ ≈ 0.

Combining (4), (5), and (6) by taking the above multiple disturbances into account, the dynamic model can be written as:

(7) $$\{\begin{array}{l} {\dot{x}}_{1}=x_{2}\\ {\dot{x}}_{2}=D_{1}x_{1}+D_{2}x_{2}+D_{3}u^{*}+D_{4}\left(t\right)\\ {\dot{x}}_{3}=x_{1} \end{array}$$

with

(8) $$D_{1}=\frac{-1}{\left(C_{e}+\Delta C\right)\left(L_{e}+\Delta L\right)}$$

(9) $$D_{2}=\frac{-\beta }{\left(C_{e}+\Delta C\right)\left(R_{Le}+\Delta R_{Le}\right)}$$

(10) $$D_{3}=\frac{\beta \left(V_{ine}+\Delta V_{in}\right)}{\left(C_{e}+\Delta C\right)\left(L_{e}+\Delta L\right)}$$

(11) $$D_{4}\left(t\right)=\frac{\beta d_{1}\left(t\right)}{C_{e}+\Delta C}+{\dot{d}}_{2}\left(t\right)-\frac{V_{refe}+\Delta V_{ref}}{\left(C_{e}+\Delta C\right)\left(L_{e}+\Delta L\right)}$$

It should be noted that multiple disturbances of the buck converter system are considered in (5) with their own boundary limitations. Therefore, the |*D*_1_| ≤ *ψ_D_*_1_, |*D*_2_| ≤ *ψ_D_*_2_, 0 < *ψ_D_*_3min_ ≤ |*D*_3_| ≤ *ψ_D_*_3max_ can be obtained from (8)–(11), and |*D*_4_(*t*)| ≤ *ψ_D_*_4_ can also be derived from (11), where *ψ_D_*_1_, *ψ_D_*_2_, *ψ_D_*_3min_, *ψ_D_*_3max_ and *ψ_D_*_4_ are boundary constants.

### 2.3. Sensor-Driver Dynamic Model

As shown in Figure 1a, it is necessary to measure the voltage signal *x*_1_ and the current signal *x*_2_, and feed back the output control signal *u* from the INTSMC with PWM to the power switch *S_W_* of the buck converter. In practical situations involving multiple disturbances, *x*_1_ can be measured using the Hall voltage sensor due to its high accuracy and reliability. In Figure 1a, the Hall voltage sensor relies on the divider resistors *R*_1_ and *R*_2_, given by *x*_1_ = *R*_1_*V_o_*/(*R*_1_ + *R*_2_) = *βV_o_*. On the other hand, *x*_2_ is measured using a Hall current sensor as *x*_2_ = *βi_c_*/*C*. Considering the discontinuity of the *u*, it is generally transmitted to *S_w_* through an isolated driver to ensure stable high-frequency switching operation. Different from conventional modeling, this paper discusses the behavior of the buck system when subjected to discontinuous control and in the presence of unmodeled dynamics. Typically, these unmodeled dynamics originate from the sensor and driver in Figure 1a, which have small time constants that are disregarded during system modeling. However, it is essential to consider these dynamics in order to examine the dynamic convergence of the state in the buck system. This issue becomes more relevant in compact microsystem platforms, where sensing circuits, driver chips, and power devices are closely integrated, and their fast but non-ideal dynamics may directly affect the voltage supplied to actuator-related modules.

**Figure 2:**
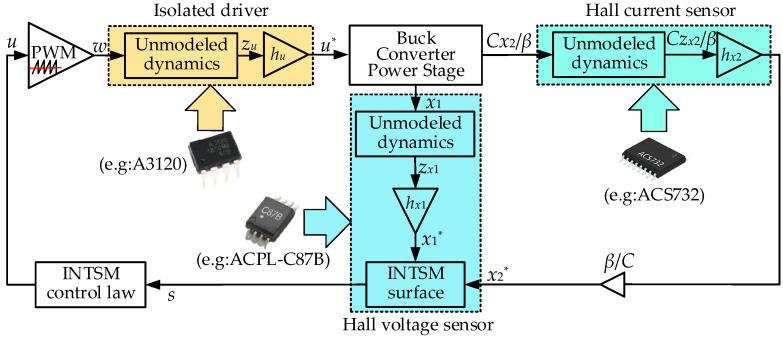
Control block of the buck converter power interface with sensor-driver dynamics.

The Hall voltage sensor is taken as an example for analysis. According to sensor theory [25], the characteristics of a real Hall voltage sensor consist of both static and dynamic components. Consequently, this paper incorporates these components into the modeling process. Additionally, to explicitly describe the signal transmission among the controller, PWM modulator, isolated driver, sensors, and converter power stage, the closed-loop structure in Figure 1a is further represented by the block diagram in Figure 2. In Figure 2, *x*_1_ represents the input state of the Hall voltage sensor, while *x*_1_* represents the output state.

**Figure 3:**
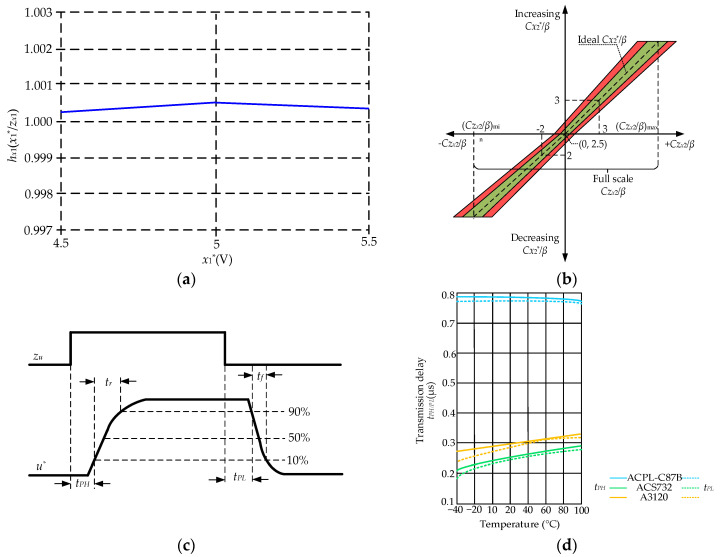
Input–output linearity characteristics of the sensor-drivers: (**a**) ACPL-C87B; (**b**) ACS732; (**c**) A3120; (**d**) transmission delay curve with temperature.

Since the Hall voltage sensors used in buck converter systems are generally linear, *h_x_*_1_ < 1 is used to describe the static linear input–output transformation between the actual voltage-related state *x*_1_ and the sensor output state $x_{1}^{*}$ as [26]:

(12) $$x_{1}^{*}=h_{x1}z_{x1}$$

where *z_x_*_1_ is the fast dynamic variable corresponding to the state *x*_1_; *h_x_*_1_ is the static linear gain. Importantly, it should be acknowledged that even with a sensor chip of high measurement accuracy, the *h_x_*_1_ can be further observed from Figure 3a.

For a buck converter system, since two types of time scales exist, singular perturbation theory is adopted to model them as [27]:

(13) $${\ddot{z}}_{x1}=-\frac{Q_{x1}}{\mu_{x1}}{\dot{z}}_{x1}-\frac{1}{\mu_{x1}^{2}}z_{x1}+\frac{1}{\mu_{x1}^{2}}P_{x1}x_{1}$$

where *Q_x_*_1_ and *P_x_*_1_ are constants satisfying *h_x_*_1_*P_x_*_1_ = 1; *μ_x_*_1_ ˂˂ 1 is a perturbation parameter. In this paper, the dynamic rise time is selected as the perturbation parameter *μ_x_*_1_, and its value can be directly obtained from the chip manual.

**Remark** **1.** *By defining Z_x*11*_ = z_x*1*_, Z_x*12*_ = μ_x*1*_* ${\dot{Z}}_{x11}$ *, (13) can be further rewritten as:*

(14) $$\{\begin{array}{l} \mu_{x1}{\dot{Z}}_{x11}=Z_{x12}\\ \mu_{x1}{\dot{Z}}_{x12}=-Q_{x1}Z_{x12}-Z_{x11}+P_{x1}x_{1} \end{array}$$

Since *x*_1_ is generated by the buck converter power-stage dynamics within |*x*_1_| ≤ *X*_1_ by the boundary of 0 ≤ *V_in_* ≤ *V_in_*_max_, its time derivative *x*_2_ is also bounded within |*x*_2_| ≤ *X*_2_ and $\left|{\dot{x}}_{2}\right|$ ≤ *X*_3_, where *X*_1_, *X*_2_ and *X*_3_ are boundary constants. Thus, the conventional singular perturbation theory [28] can apply to (14), which implies that after a finite time interval Δ*t* (Δ*t* → 0 with *μ_x_*_1_ → 0), variable *z_x_*_1_ converges into a small vicinity of *P_x_*_1_*x*_1_, i.e.,

(15) $$x_{1}^{*}=h_{x1}z_{x1}=h_{x1}P_{x1}x_{1}+\varepsilon_{x1}\left(\mu_{x1}\right)=x_{1}+\varepsilon_{x1}\left(\mu_{x1}\right)$$

where *ε_x_*_1_(*μ_x_*_1_) tends to zero as *μ_x_*_1_ → 0.

**Remark** **2.** Figure 3 shows the input/output characteristics of sensor-drivers in three different ways. For the Hall current sensor shown in Figure 2, the output signal associated with the static linear gain h_x2_ can be expressed as C*x_2_*/β*. However, according to Figure 3b, there is a strong linear relationship between the output and input signals of the Hall current sensor. Therefore, for convenience of analysis, the transmission dynamics of C*/β* can be further superimposed on x_2_*, and the output characteristics, as shown in Figure 2, can be obtained.

For the isolated driver in Figure 3c, it can be seen that there is also a strong linear relationship between the input and output signals. However, the signal transmission delay *t_P_* is worth paying attention to in the dynamic characteristics of the device. However, for the typical isolated driver represented by A3120, the range of *t_P_* with temperature variation is between 0.25 and 0.32 μs, as shown in Figure 3d, which can be ignored compared with the dynamic response of the whole buck system at the millisecond level. Similarly, the transmission delay of the Hall voltage sensor ACPL-C87B and Hall current sensor ACS732 can also be ignored.

According to (15), the input–output characteristics of the Hall current sensor and isolated driver in Figure 2 can be obtained as (16) and (17), respectively:

(16) $$x_{2}^{*}=h_{x2}z_{x2}$$

(17) $$u^{*}=h_{u}z_{u}$$

where *z_x_*_2_ and *z_u_* are the fast dynamic variables corresponding to the states *x*_2_ and *x_u_*, respectively; *h_x_*_2_ and *h_u_* are the static linear gains shown in Figure 3; the superscript “*” denotes the signal available to the controller or applied to the power switch after the sensor/driver dynamics.

Referring to (14), the state-space form of *x*_2_* can be given from (16) as:

(18) $$\{\begin{array}{l} Z_{x21}=z_{x2}\\ {\dot{Z}}_{x21}=\frac{Z_{x22}}{\mu_{x2}}\\ {\dot{Z}}_{x22}=-\frac{Q_{x2}Z_{x22}}{\mu_{x2}}-\frac{Z_{x21}}{\mu_{x2}}+\frac{P_{x2}x_{2}}{\mu_{x2}} \end{array}$$

(19) $${\mu_{u}}^{2}{\ddot{z}}_{u}+\mu_{u}Q_{u}{\dot{z}}_{u}+z_{u}=P_{u}w$$

where *Q_x_*_2_, *P_x_*_1_, *Q_u_*, and *P_u_* are constants that satisfy *h_x_*_2_*P_x_*_2_ = *h_u_P_u_* = 1; *μ_x_*_2_ and *μ_u_* are perturbation parameters, which can usually be adopted as the dynamic rise time *t_PLH_* in Figure 3 and remain in the same order of magnitude as *μ_x_*_1_. For the system state *x*_2_ bounded by *X*_2_ and the discontinuous control law *u*, there is also a finite time interval Δ*t* (Δ*t* → 0 with *μ_x_*_2_ → 0 and *μ_u_* → 0) that variables *Z_x_*_21_ and *Z_u_* can converge into a small vicinity of *P_x_*_2_*x*_2_ and *P_u_u*, which can be described as:

(20) $$x_{2}^{*}=h_{x2}P_{x2}x_{2}+\varepsilon_{x2}\left(\mu_{x2}\right)=x_{2}+\varepsilon_{x2}\left(\mu_{x2}\right)$$

(21) $$u^{*}=h_{u}P_{u}u+\varepsilon_{u}\left(\mu_{u}\right)=w+\varepsilon_{u}\left(\mu_{u}\right)$$

where neighbors *ε_x_*_2_(*μ_x_*_2_) and *ε_u_*(*μ_u_*) tend to zero as (*μ_x_*_2_, *μ_u_*) → (0, 0).

It can be easily seen from (15) and (20) that the sensor output signal ${{\dot{x}}_{1}}^{*}$ = *x*_2_* can still inherit the differential characteristics of the input signal *x*_1_* outside the small vicinity. Hence, the time-varying signal ${{\dot{x}}_{2}}^{*}$ can be further expressed based on the system dynamic model (7) as

(22) $${\dot{x}}_{2}^{*}={\dot{x}}_{2}+\varepsilon_{\dot{x}2}\left(\mu_{x1},\mu_{x2},\mu_{u}\right)$$

where the neighbors $\varepsilon_{{\dot{x}}_{2}}$(*μ_x_*_1_, *μ_x_*_2_, *μ_u_*) tend to zero as (*μ_x_*_1_, *μ_x_*_2_, *μ_u_*) → (0, 0, 0).

**Remark** **3.** *To determine the model parameters (h_x*1*_, h_x*2*_, h_u_), (P_x*1*_, P_x*2*_, P_u_) and (Q_x*1*_, Q_x*2*_, Q_u_) in (12), (14), (18) and (19), it is recommended to carry out model identification and fitting based on the test data of sensor and driver chips, and the implementation process is illustrated in the simulation section. It is simple, accurate, and close to the real performance of the real Hall sensors and the isolated driver.*

## 3. Design and Robust Stability Analysis of INTSMC Under Multi-Source Disturbances and Sensor-Driver Dynamics

Based on the control-oriented model derived in Section 2, the objective of this section is to design a PWM-realizable INTSMC law such that the output voltage can track the reference value under multi-source disturbances and sensor-driver dynamics. In micro-actuator power interfaces, this objective is closely related to the suppression of voltage deviation and current fluctuation during rapid actuator-side command or load changes.

### 3.1. Design of the INTSMC Controller

**Figure 4:**
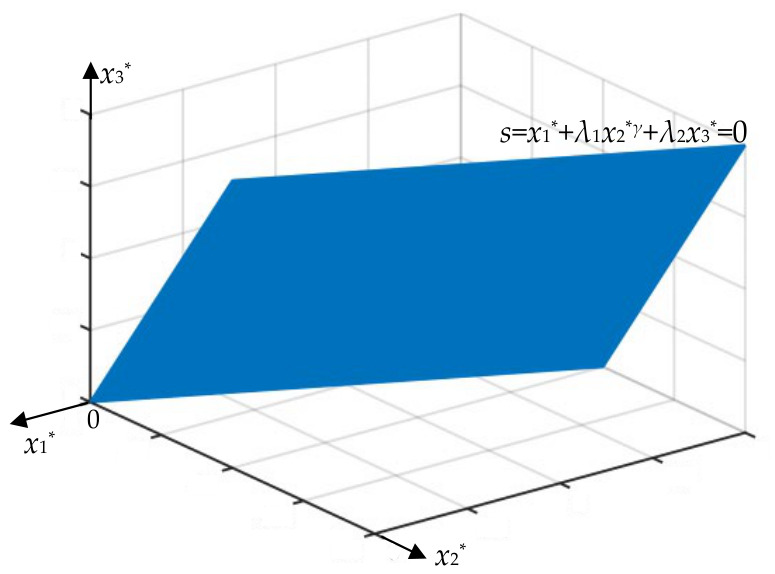
Graphical representation of the sliding surface in 3D space.

Based on the principle of NTSM [29], the controller design consists of a sliding surface *s*, which can describe the dynamic characteristics of the arbitrary system state in the phase plane, and a robust control law u* determining the switching operation of the system. Firstly, the INTSMC surface *s* can be designed as:

(23) $$s={x_{1}}^{*}+\lambda_{1}x_{2}^{*\gamma }+\lambda_{2}{x_{3}}^{*}$$

where *λ*_1_ and *λ*_2_ represent the positive control parameters termed as sliding coefficients; *γ* = *p*/*q*, where *q* and *p* are positive odd integers satisfying 0 < *q* < *p* < 2*q*. And the additional voltage error integral term is included as a state variable of the controller to reduce the steady-state error of the system. The graphical representation of the designed sliding surface in three-dimensional (3-D) space is illustrated in Figure 4.

**Theorem** **1.** *The sliding manifold (23) and the following control laws ensure the asymptotic convergence of the voltage error dynamics (4):*

(24) $$u_{eq}=\frac{-1}{\lambda_{1}\gamma D_{3}}\left(\lambda_{1}\gamma D_{1}{x_{1}}^{*}+\lambda_{1}\gamma D_{2}{x_{2}}^{*}+x_{2}^{*2-\gamma }+\lambda_{2}{x_{1}}^{*}x_{2}^{*1-\gamma }+\lambda_{1}\gamma D_{4}\left(t\right)\right)$$

(25) $$u_{n}=-M\mathrm{sgn}\left(s\right)$$

*where u_eq_ is the equivalent control term which drives the system state points to reach the origin along the sliding surface s = *$\dot{s}$* = 0; u_n_ is a switching control term that attracts the system state points to s = 0 to complete the approaching motion; M > 0 is the switching control gain. It should also be noted that the discontinuous u_n_ and continuous u_eq_ together constitute the duty ratio of PWM in* *Figure 1a.*

(26) $$u=u_{eq}+u_{n}$$

**Remark** **4.** *It is easy to understand the PWM modulation of continuous u_eq_ in the approach motion of the INTSMC. For the discontinuous u_n_ in the sliding motion, in terms of a PWM-based controlled system, the instantaneous duty cycle u can be expressed as:*

(27) $$u=\frac{\left|u_{n}\right|}{\left|V_{ramp}\right|}=\frac{M}{{\hat{V}}_{ramp}}$$

*where* ${\dot{V}}_{ramp}$* is the peak magnitude of the ramp single V_ramp_. Since u ∈ [0, 1], the selection of switching control gain M should be considered in combination with the amplitude of V_ramp_, and M should also be limited to the range of system stability conditions, which will be further discussed later.*

In Figure 1a, the obtained control signal *w* from PWM should satisfy *w* = 0.5[1 + sgn(*s*)] based on the characteristics of the control signal u* in system model (3), and *w* can effectively be a duty cycle control of *u* at a high switching frequency of PWM [30]. Therefore, only *u* is quoted to describe the INTSMC system later.

It should be noted that the PWM-realizable form in (24)–(27) does not imply ideal chattering-free sliding motion. Since the switching term *u_n_* in (25) is discontinuous, the fixed-frequency PWM implementation generates a quasi-sliding band around the INTSMC surface *s* = 0 in (23). According to the driver model in (17), (19), and (21), the effective switching action can be expressed as u* = *h_u_z_u_* = *w* + *ε_u_*(*μ_u_*), where *w* is the PWM signal before the isolated driver, *z_u_* is the fast driver dynamic variable, and *ε_u_*(*μ_u_*) is the driver-induced deviation. Therefore, the finite bandwidth and propagation delay of the driver may reshape the high-frequency component of www, enlarge the quasi-sliding band, and increase the local ripple of *V_o_*, *i_L_*, and *s*.

For compact micro-actuator power networks, excessive residual chattering may increase switching loss, voltage/current stress on *S_W_*, and conducted electromagnetic noise. Therefore, *M* should be coordinated with *V_ramp_*, the sampling period, the PWM frequency, and the driver response parameter *μ_u_*, rather than being selected only for faster reaching. In practical implementation, sgn(*s*) can be replaced by the boundary-layer approximation sat(*s*/*ϕ*), and the implemented duty ratio can be constrained as *u* = sa_t [0,1]_(*u_eq_* − *M*sat(*s*/*ϕ*)), where *ϕ* > 0 is the boundary-layer width. This treatment reduces residual chattering and switch stress at the cost of a small steady-state neighborhood.

**Proof** **of Theorem 1.** Differentiating (21) with respect to time *t* gives the following:

(28) $$\dot{s}={x_{2}}^{*}+\lambda_{1}\gamma x_{2}^{*\gamma -1}{{\dot{x}}_{2}}^{*}+\lambda_{2}{x_{1}}^{*}$$

Substituting (7), (15), (20), and (22) into (28) gives the following:

(29) $$\dot{s}=\lambda_{1}\gamma x_{2}^{*\gamma -1}\left[D_{1}{x_{1}}^{*}+D_{2}{x_{2}}^{*}+D_{3}\left(u_{eq}+u_{n}\right)+D_{4}\left(t\right)+\frac{1}{\lambda_{1}\gamma }x_{2}^{*2-\gamma }+\frac{\lambda_{2}}{\lambda_{1}\gamma }{x_{1}}^{*}x_{2}^{*1-\gamma }\right]-\varepsilon_{s}\left(\mu_{x1},\mu_{x2},\mu_{u}\right)$$

where

(30) $$\varepsilon_{s}\left(\mu_{x1},\mu_{x2},\mu_{u}\right)=\max\left\{\varepsilon_{x1}\left(\mu_{x1}\right),\;\varepsilon_{x2}\left(\mu_{x2}\right),\;\varepsilon_{\dot{x}2}\left(\mu_{x1},\mu_{x2},\mu_{u}\right)\right\}$$

where the neighbor *ε_s_*(*μ_x_*_1_, *μ_x_*_2_, *μ_u_*) tends to zero as (*μ_x_*_1_, *μ_x_*_2_, *μ_u_*) → (0, 0, 0).

To prove that *βV_o_* tracks *V_ref_*, consider the candidate Lyapunov function *V* = 0.5 *s*^2^. Taking its derivative along the trajectories of the dynamics (7), it follows that:

(31) $$\begin{array}{cc} \dot{V} & =s\dot{s}\\ & =s\left(\lambda_{1}\gamma x_{2}^{*\gamma -1}\right)\left[D_{1}{x_{1}}^{*}+D_{2}{x_{2}}^{*}+D_{3}\left(u_{eq}+u_{n}\right)+D_{4}\left(t\right)+\frac{1}{\lambda_{1}\gamma }x_{2}^{*2-\gamma }+\frac{\lambda_{2}}{\lambda_{1}\gamma }{x_{1}}^{*}x_{2}^{*1-\gamma }\right]-\varepsilon_{s}\left(\mu_{x1},\mu_{x2},\mu_{u}\right) \end{array}$$

Substituting (25) into the above yields the following:

(32) $$\dot{V}\leq s\left(\lambda_{1}\gamma x_{2}^{*\gamma -1}\right)\left[\left|D_{1}{x_{1}}^{*}+D_{2}{x_{2}}^{*}+D_{3}u_{eq}+D_{4}\left(t\right)+\frac{1}{\lambda_{1}\gamma }x_{2}^{*2-\gamma }+\frac{\lambda_{2}}{\lambda_{1}\gamma }{x_{1}}^{*}x_{2}^{*1-\gamma }\right|\right]-\left(M\lambda_{1}\gamma x_{2}^{*\gamma -1}\right)\left|s\right|-\varepsilon_{s}\left(\mu_{x1},\mu_{x2},\mu_{u}\right)$$

Further substituting (24) into the above gives the following:

(33) $$\dot{V}\leq -\left(M\lambda_{1}\gamma x_{2}^{*\gamma -1}\right)\left|s\right|-\varepsilon_{s}\left(\mu_{x1},\mu_{x2},\mu_{u}\right)=-\eta \left(x_{2}^{*}\right)V^{\frac{1}{2}}-\varepsilon_{s}\left(\mu_{x1},\mu_{x2},\mu_{u}\right)$$

where *η*(*x*_2_*) = *Mλ*_1_*γx*_2_*^γ^^−1^ > 0.

**Figure 5:**
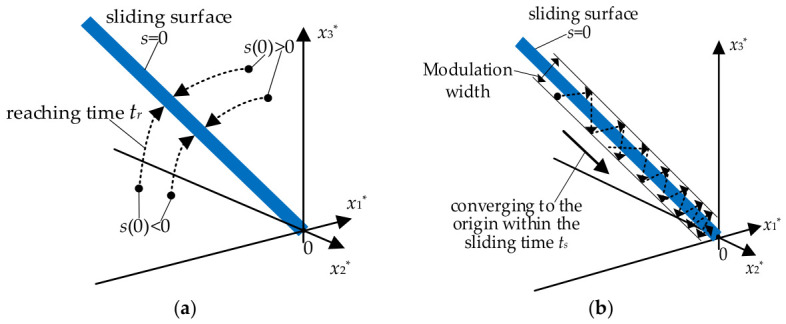
Graphical representations of system states PT’s behavior in INTSMC control process: (**a**) Phase 1; (**b**) Phase 2.

When the system state has not reached the sliding surface, i.e., *s* ≠ 0, because of 0 < (*γ* − 1) < 1 and *p* and *q* are positive odd integers in (23), there is *x*_2_*^γ^^−1^ > 0 for any *x*_2_* ≠ 0, and *x*_2_*^γ^^−1^ = 0 for *x*_2_* = 0. Therefore, two different cases are considered for *s* ≠ 0:

(1) If *x*_2_* ≠ 0, the $\dot{V}$ < 0 can be held in (33), which means that system voltage error dynamics (7) will reach *s* = 0 in a finite arrival time *t_r_* by the control law *u_n_*, where *s*(0) = (*x*_1_*(0), *x*_2_*(0)) is the initial state of the system. And the analysis will be given in Section 4. And this stage is phase 1 of the entire state convergence process caused by the INTSMC control system, which is graphically demonstrated in Figure 5a. On *s* = 0, it can be obtained from (15) as follows:

(34) $${x_{1}}^{*}+\lambda_{1}{{\dot{x}}_{1}}^{*\gamma }+\lambda_{2}\int_{0}^{t_{r}}{x_{1}}^{*}dt=0$$

which means that the system voltage error *x*_1_* will converge to zero asymptotically along *s* = 0 after *s* reaches zero by the control law *u_eq_*. This phase corresponds to the convergence phase 2 of the INTSMC system, which is further described in Figure 5b.

**Figure 6:**
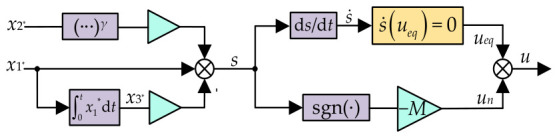
Block diagram of INTSMC for buck converters.

(2) If *x*_2_* = ${{\dot{x}}_{1}}^{*}$ = 0, since *s* ≠ 0, from (28), there must be *x*_1_* ≠ 0, and it can easily be proved that the system will not always stay on the point (${{\dot{x}}_{1}}^{*}$ = 0, *x*_1_* ≠ 0), and the possible points satisfying $\dot{V}$ = 0 are isolated and transient; once the system state point escapes from this unstable state, $\dot{V}$ < 0 will be satisfied again. Therefore, the system dynamics can reach the INTSMC sliding surface *s* = 0 inside the *ε_s_*(*μ_x_*_1_, *μ_x_*_22_, *μ_u_*)-vicinity within the finite reaching time *t_r_*, and finally converge to the origin within the sliding time *t_s_*, as Figure 5 describes. This completes the proof. The INTSMC block diagram of the buck converter is shown in Figure 6. This completes the proof of Theorem 1. □

**Remark** **5.** In the equivalent control term (24), it is obvious that as long as 1 < γ < 2, the term x_2_*^γ−1^ is always non-singular. Therefore, the singularity problem will not occur in the INTSMC with the control law in (24)–(26).

### 3.2. Existence Condition Analysis of the System’s Control Parameters and Disturbance

(33) gives the SMC stability condition of the INTSMC system, which is closely related to the parameters *λ*_1_ and *λ*_2_ of the control system and disturbance terms *D*_1_, *D*_2_, *D*_3_, and *D*_4_(*t*). Considering that the *s* = 0 and *s* ≠ 0 cases of the Lyapunov function *V* = 0.5 *s*^2^ have been discussed before and after (34), the influence of control parameters and disturbance terms on INTSMC system stability will be analyzed by further supplementing the cases of *s* > 0 and *s* < 0.

Further combining with (29), it can be divided into the cases of *s* > 0 and *s* < 0 as:

(35) $$l_{1}:\;\dot{s}={x_{2}}^{*}+\lambda_{2}{x_{1}}^{*}+\lambda_{1}\gamma x_{2}^{*\gamma -1}[D_{1}{x_{1}}^{*}+D_{2}{x_{2}}^{*}+D_{4}\left(t\right)-\varepsilon_{s}\left(\mu_{x1},\mu_{x2},\mu_{u}\right)]<0,\;s>0,\;u=0$$

(36) $$l_{2}:\;\dot{s}={x_{2}}^{*}+\lambda_{2}{x_{1}}^{*}+\lambda_{1}\gamma x_{2}^{*\gamma -1}\left[D_{1}{x_{1}}^{*}+D_{2}{x_{2}}^{*}+D_{3}+D_{4}\left(t\right)-\varepsilon_{s}\left(\mu_{x1},\mu_{x2},\mu_{u}\right)\right]>0,\;s<0,\;u=1$$

**Figure 7:**
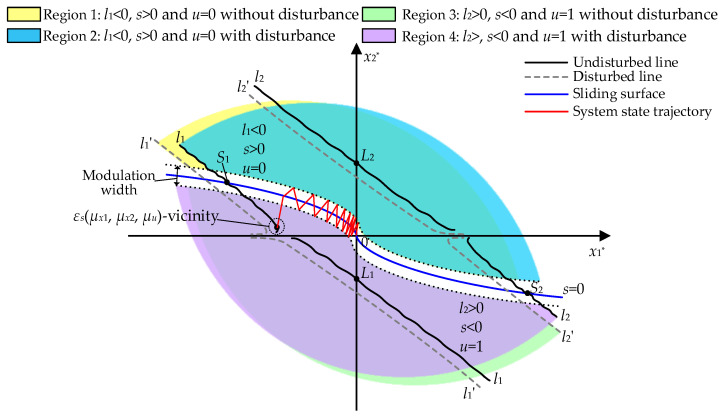
Regions of existence of the INTSMC under multi-source disturbances.

The characteristics of the system parameters in (35) and (36) can be combined to determine the different regions of existence of the INTSMC in Figure 7. The sliding surface *s* = 0 splits the phase plane into two regions. In each region, the state PT is directed toward *s* = 0 by an appropriate switching action. The sliding mode occurs only on the portion of *s* = 0 that covers both regions. This portion is within points *S*_1_ and *S*_2_.

Combining (35) and (36) gives

(37) $$\begin{array}{cc} l-\lambda_{1}\gamma x_{2}^{*\gamma -1}\left[D_{1}{x_{1}}^{*}+D_{2}{x_{2}}^{*}+D_{3}+D_{4}\left(t\right)-\varepsilon_{s}\left(\mu_{x1},\mu_{x2},\mu_{u}\right)\right] & <{x_{2}}^{*}+\lambda_{2}{x_{1}}^{*}\\ & <-\lambda_{1}\gamma x_{2}^{*\gamma -1}\left[D_{1}{x_{1}}^{*}+D_{2}{x_{2}}^{*}+D_{4}\left(t\right)-\varepsilon_{s}\left(\mu_{x1},\mu_{x2},\mu_{u}\right)\right] \end{array}$$

It can easily be shown that the existence condition $\dot{V}$ < 0 is guaranteed if the following condition holds:

(38) $$-D_{3}<\frac{x_{2}^{*1-\gamma }}{\lambda_{1}\gamma }\left({x_{2}}^{*}+\lambda_{2}{x_{1}}^{*}\right)+D_{1}{x_{1}}^{*}+D_{2}{x_{2}}^{*}+D_{4}\left(t\right)-\varepsilon_{s}\left(\mu_{x1},\mu_{x2},\mu_{u}\right)<0$$

Note that it is very difficult to find the regions of system control parameters and disturbance variables defined by the inequality in (38). However, it can be shown that the INTSMC exists around the equilibrium point (*x*_1_* ≈ 0, *x*_2_* ≈ 0), if the following condition is satisfied:

(39) $$-D_{3}<D_{4}\left(t\right)-\varepsilon_{s}\left(\mu_{x1},\mu_{x2},\mu_{u}\right)<0$$

Further substituting (10) and (11) into (39), the existence condition can be further expressed as

(40) $$\beta \left(V_{ine}+\Delta V_{in}\right)>V_{refe}+\Delta V_{ref}-\beta d_{1}\left(t\right)\left(L_{e}+\Delta L\right)-\left(L_{e}+\Delta L\right)\left(C_{e}+\Delta C\right){\dot{d}}_{2}\left(t\right)+\varepsilon_{s}\left(\mu_{x1},\mu_{x2},\mu_{u}\right)>0$$

(40) gives the existence conditions of the designed INTSMC system under the disturbance of the internal parameters of the buck converter. It can be seen that the input voltage range 0 ≤ *V_in_* ≤ *V_in_*_max_ in Figure 1 plays a decisive role in the stability of the whole system.

It can be seen from Figure 7 that the slope of these lines is negative. Note that this changes the *x*_2_ intercepts of the lines *l*_1_ and *l*_2_ to *L*_1_ and *L*_2_, respectively. The dynamic response of the buck converter depends on the value of the control parameter *λ*_l_ and disturbance terms *D*_2_, *D*_3_, and *D*_4_(*t*). To ensure the dynamic response and sufficient existence region, the relationship between the above system variables can be described as:

(41) $$\frac{1}{4\lambda_{1}\gamma D_{2}{\left[{\lambda_{1}}^{2}\gamma^{2}D_{2}D_{4}\left(t\right)\right]}^{\frac{2-\gamma }{2\gamma -3}}}-\frac{\lambda_{1}\gamma D_{4}\left(t\right)}{\lambda_{1}\gamma D_{2}}<0<\frac{1}{4\lambda_{1}\gamma D_{2}{\left[{\lambda_{1}}^{2}\gamma^{2}D_{2}\left(D_{3}+D_{4}\left(t\right)\right)\right]}^{\frac{2-\gamma }{2\gamma -3}}}-\frac{\lambda_{1}\gamma \left(D_{3}+D_{4}\left(t\right)\right)}{\lambda_{1}\gamma D_{2}}$$

**Remark** **6.** *The neighbor group [ε_x_*_1_ *(μ_x_*_1_ *), ε_x_*_2_ *(μ_x_*_2_ *), ε_u_(μ_u_)]^T^ tends to zero with [μ_x_*_1_ *, μ_x_*_2_ *, μ_u_]^T^ → [0, 0, 0]* *^T^, and the characteristics of [μ_x_*_1_ *, μ_x_*_2_ *, μ_u_]^T^ << [1, 1, 1]* *^T^ further guarantee that [ε_x_*_1_ *(μ_x_*_1_ *), ε_x_*_2_ *(μ_x_*_2_ *), ε_u_(μ_u_)]^T^ << [1, 1, 1]* *^T^. Therefore, for convenience of expression, the linear terms containing ε_x_*_1_ *(μ_x_*_1_ *), ε_x_*_2_ *(μ_x_*_2_ *), or ε_u_(μ_u_) can be equivalent to each other in the theoretical calculation process, and the integrity of the dynamic model of the system sensor-driver dynamics can still be ensured.*

### 3.3. Existence Condition Analysis of System Sensor-Driver Dynamics

Combining Remark 3, the significance of *h_x_*_1_*P_x_*_1_ = *h_x_*_2_*P_x_*_2_ = *h_u_P_u_* = 1 lies in the choice and regulation of real Hall sensors and the isolated driver in practical applications.

For the buck system in Figure 1, although the stability of system states considering sensor-driver dynamics beyond the vicinity *ε_s_*(*μ_x_*_1_, *μ_x_*_2_, *μ_u_*) of *s* = 0 has been analyzed, the analysis of the control law *u* still needs to be demonstrated by the strict sliding mode stability theorem. Then, from (28), the second and the third time derivatives of the sliding variable *s*_1_ = *s* may be further found by combining (7), (14), (18), and (19) as:

(42) $$s_{2}={\dot{s}}_{1}=\frac{1}{\mu_{x1}\mu_{x2}}\left(\mu_{x2}h_{x1}Z_{x12}+\mu_{x1}\lambda_{1}\gamma {h_{x2}}^{\gamma }{Z_{x21}}^{\gamma -1}Z_{x22}+\mu_{x1}\mu_{x2}\lambda_{2}h_{x1}Z_{x11}\right)$$

(43) $$\begin{array}{ccc} s_{3} & ={\dot{s}}_{2} & \\ & =\frac{1}{{\left(\mu_{x1}\mu_{x2}\right)}^{2}} & [-{\mu_{x2}}^{2}h_{x1}Q_{x1}Z_{x12}-{\mu_{x2}}^{2}h_{x1}Z_{x11}+{\mu_{x2}}^{2}h_{x1}P_{x1}x_{1}+{\mu_{x1}}^{2}\lambda_{1}\gamma \left(\gamma -1\right){h_{x2}}^{\gamma }{Z_{x21}}^{\gamma -2}{Z_{x22}}^{2}\\ & & -{\mu_{x1}}^{2}\lambda_{1}\gamma Q_{x2}{h_{x2}}^{\gamma }{Z_{x21}}^{\gamma -1}Z_{x22}-{\mu_{x1}}^{2}\lambda_{1}\gamma {h_{x2}}^{\gamma }{Z_{x21}}^{\gamma }+{\mu_{x1}}^{2}\lambda_{1}\gamma P_{x2}{h_{x2}}^{\gamma }{Z_{x21}}^{\gamma -1}x_{2}\\ & & +\mu_{x1}{\mu_{x2}}^{2}\lambda_{2}h_{x1}Z_{x12}] \end{array}$$

(44) $${\dot{s}}_{3}=\frac{1}{{\mu_{x2}}^{2}}{Z_{x21}}^{\gamma -1}\left\{H\left(x,Z_{1},Z_{2}\right)-M\lambda_{1}\gamma P_{x2}{h_{x2}}^{\gamma }D_{3}\mathrm{sgn}\left(s\right)\right\}$$

(45) $$\begin{array}{cc} H\left(x,Z_{1},Z_{2}\right)= & \{\frac{{\mu_{x2}}^{2}}{{\mu_{x1}}^{3}}h_{x1}\left(Q_{x1}-\mu_{x1}\lambda_{2}\right)\frac{Z_{x11}}{{Z_{x21}}^{\gamma -1}}-\frac{{\mu_{x2}}^{2}}{{\mu_{x1}}^{3}}h_{x1}\left(1+{\mu_{x1}}^{2}\lambda_{2}Q_{x1}-{Q_{x1}}^{2}\right)\frac{Z_{x12}}{{Z_{x21}}^{\gamma -1}}\\ & +\frac{{\mu_{x2}}^{2}}{{\mu_{x1}}^{3}}\left(\mu_{x1}\lambda_{2}-Q_{x1}\right)\frac{x_{1}}{{Z_{x21}}^{\gamma -1}}+\frac{{\mu_{x2}}^{2}}{{\mu_{x1}}^{2}}\frac{x_{2}}{{Z_{x21}}^{\gamma -1}}+\frac{1}{\mu_{x2}}\lambda_{1}\gamma Q_{x2}{h_{x2}}^{\gamma }Z_{x21}\\ & -\frac{1}{\mu_{x2}}\lambda_{1}\gamma {h_{x2}}^{\gamma }\left(3\gamma -2-{Q_{x2}}^{2}\right)Z_{x22}-3\lambda_{1}\gamma \left(\gamma -1\right){h_{x2}}^{\gamma }Q_{x2}\frac{{Z_{x22}}^{2}}{Z_{x21}}\\ & -\frac{1}{\mu_{x2}}\lambda_{1}\gamma \left(\gamma -1\right)\left(2-\gamma \right){h_{x2}}^{\gamma }\frac{{Z_{x22}}^{3}}{{Z_{x21}}^{2}}-\frac{1}{\mu_{x2}}\lambda_{1}\gamma P_{x2}{h_{x2}}^{\gamma }Q_{x2}x_{2}\\ & -P_{x2}{h_{x2}}^{\gamma }x_{2}^{2-\gamma }-\lambda_{2}P_{x2}{h_{x2}}^{\gamma }x_{1}x_{2}^{1-\gamma }+\frac{3}{\mu_{x2}}\lambda_{1}\gamma \left(\gamma -1\right)P_{x2}{h_{x2}}^{\gamma }\frac{Z_{x22}x_{2}}{Z_{x21}}+\varepsilon_{H}\left(\mu_{x1},\mu_{x2},\mu_{u}\right)\} \end{array}$$

(46) $$\varepsilon_{H}\left(\mu_{x1},\mu_{x2},\mu_{u}\right)=\max\left\{\varepsilon_{x1}\left(\mu_{x1}\right),\varepsilon_{x2}\left(\mu_{x2}\right),\varepsilon_{u}\left(\mu_{u}\right)\right\}$$

where ***Z***_1_ = [*Z_x_*_11_, *Z_x_*_12_]^T^, ***Z***_2_ = [*Z_x_*_21_, *Z_x_*_22_]^T^, and *ε_H_*(*μ_x_*_1_, *μ_x_*_2_, *μ_u_*) → 0 as (*μ_x_*_1_, *μ_x_*_2_, *μ_u_*) → (0, 0, 0).

By combining multiple derivatives of the sliding variable *s*, a Lyapunov-like function V* can be designed as follows:

(47) $$V^{*}=s_{1}s_{3}-\frac{1}{2}{s_{2}}^{2}$$

and it is noted that the function (47) is sign-varying. Then the time derivative of the function V* can be written as:

(48) $$\begin{array}{ll} {\dot{V}}^{*} & =s_{1}{\dot{s}}_{3}\\ & =\frac{1}{{\mu_{x2}}^{2}}{Z_{x21}}^{\gamma -1}\left\{H\left(x,Z_{1},Z_{2}\right)s-M\lambda_{1}\gamma P_{x2}{h_{x2}}^{\gamma }D_{3}\left|s\right|\right\}\\ & \leq \frac{1}{{\mu_{x2}}^{2}}\left|s\right|{Z_{x21}}^{\gamma -1}\left\{\left|H\left(x,Z_{1},Z_{2}\right)\right|-M\lambda_{1}\gamma \psi_{D3\min}\right\}\\ & \leq \frac{1}{{\mu_{x2}}^{2}}\left|s\right|{Z_{x21}}^{\gamma -1}\left(\psi_{H}-M\lambda_{1}\gamma \psi_{D3\min}\right) \end{array}$$

where *ψ_H_* > 0 is the boundary of *H*(***x***, ***Z***_1_, ***Z***_2_). From (20), there is *ε_x_*_2_(*μ_x_*_2_) → 0 as *μ_x_*_2_ → 0, *Zx*_21_ ≠ 0 can be guaranteed in (45) and (48). By virtue of the uniqueness of *γ* − 1, the stability condition of the INTSMC system can be obtained from (48) as

(49) $$M\lambda_{1}\gamma >\frac{\psi_{H}}{\psi_{D3\min}}$$

**Remark** **7.** *Considering that the multinomial property of H(x, Z_1_, Z_2_) in (49) is not conducive to the fast determination of boundary ψ_H_, an estimation method of ψ_H_ is given to simplify the calculation here.*

By combining (15) and (20) with (45), *H*(***x***, ***Z***_1_, ***Z***_2_) can be equivalent to the following:

(50) $$\begin{array}{cc} H\left(x,Z_{1},Z_{2}\right) & \overset{\lim\left(\mu_{x2},\mu_{x2}\right)\to \left(0,0\right)}{\to }\\ & \{\frac{1}{\mu_{x1}}h_{x1}\left(Q_{x1}-\mu_{x1}\lambda_{2}\right)\frac{Z_{x11}}{{Z_{x21}}^{\gamma -1}}-\frac{1}{\mu_{x1}}\left[h_{x1}Q_{x1}\left({\mu_{x1}}^{2}\lambda_{2}-Q_{x1}\right)+h_{x1}\right]\frac{Z_{x12}}{{Z_{x21}}^{\gamma -1}}+\frac{1}{\mu_{x1}}\left(\mu_{x1}\lambda_{2}-Q_{x1}\right)\frac{x_{1}}{{Z_{x21}}^{\gamma -1}}\\ & +\left(\frac{1}{{Z_{x21}}^{\gamma -1}}-\frac{1}{\mu_{x2}}\lambda_{1}\gamma P_{x2}{h_{x2}}^{\gamma }Q_{x2}\right)x_{2}+\frac{1}{\mu_{x2}}\lambda_{1}\gamma Q_{x2}{h_{x2}}^{\gamma }Z_{x21}-\frac{1}{\mu_{x2}}\lambda_{1}\gamma {h_{x2}}^{\gamma }\left(3\gamma -2-{Q_{x2}}^{2}\right)Z_{x22}\\ & -3\lambda_{1}\gamma \left(\gamma -1\right){h_{x2}}^{\gamma }Q_{x2}\frac{{Z_{x22}}^{2}}{Z_{x21}}-\frac{1}{\mu_{x2}}\lambda_{1}\gamma \left(\gamma -1\right)\left(2-\gamma \right){h_{x2}}^{\gamma }\frac{{Z_{x22}}^{3}}{{Z_{x21}}^{2}}-\lambda_{2}P_{x2}{h_{x2}}^{\gamma }x_{1}x_{2}^{1-\gamma }\\ & +\frac{1}{\mu_{x2}}3\lambda_{1}\gamma \left(\gamma -1\right){h_{x2}}^{\gamma }Z_{x22}+\max\left\{\varepsilon_{x1}\left(\mu_{x1}\right),\varepsilon_{x2}\left(\mu_{x2}\right),\varepsilon_{u}\left(\mu_{u}\right)\right\}\} \end{array}$$

Since parameters *μ_x_*_1_ and *μ_x_*_2_ are selected as the rise time *t_PLH_* in practice, it can be obviously seen from Figure 3d that *μ_x_*_1_ and *μ_x_*_1_ ∈ [10^−7^, 10^−6^]; therefore, (50) can be further simplified as

(51) $$H\left(x,Z_{1},Z_{2}\right)\approx \left\{-\frac{1}{\mu_{x1}}h_{x1}\left(1-{Q_{x1}}^{2}\right)\frac{Z_{x12}}{{Z_{x21}}^{\gamma -1}}-\frac{1}{\mu_{x2}}\lambda_{1}\gamma {h_{x2}}^{\gamma }\left(1-{Q_{x2}}^{2}\right)Z_{x22}-\frac{1}{\mu_{x2}}\lambda_{1}\gamma \left(\gamma -1\right)\left(2-\gamma \right){h_{x2}}^{\gamma }\frac{{Z_{x22}}^{3}}{{Z_{x21}}^{2}}\right\}$$

Further substituting the system state boundary values |*x*_1_| ≤ *X*_1_, |*x*_2_| ≤ *X*_2_ and $\left|{\dot{x}}_{2}\right|$ ≤ *X*_3_ into (51), the estimation value of *ψ_H_* can be obtained as:

(52) $$\begin{array}{cc} \psi_{H} & \geq \left|-\frac{1}{\mu_{x1}}h_{x1}\left(1-{Q_{x1}}^{2}\right)\frac{Z_{x12}}{{Z_{x21}}^{\gamma -1}}-\frac{1}{\mu_{x2}}\lambda_{1}\gamma {h_{x2}}^{\gamma }\left(1-{Q_{x2}}^{2}\right)Z_{x22}-\frac{1}{\mu_{x2}}\lambda_{1}\gamma \left(\gamma -1\right)\left(2-\gamma \right){h_{x2}}^{\gamma }\frac{{Z_{x22}}^{3}}{{Z_{x21}}^{2}}\right|\\ & \geq \left|\frac{1}{\mu_{x1}}h_{x1}\left(1-{Q_{x1}}^{2}\right)\frac{X_{1}}{{X_{2}}^{\gamma -1}}-\frac{1}{\mu_{x2}}\lambda_{1}\gamma {h_{x2}}^{\gamma }\left(1-{Q_{x2}}^{2}\right)X_{3}-\frac{1}{\mu_{x2}}\lambda_{1}\gamma \left(\gamma -1\right)\left(2-\gamma \right){h_{x2}}^{\gamma }\frac{{X_{3}}^{3}}{{X_{2}}^{2}}\right| \end{array}$$

## 4. Phase Trajectory Analysis of the INTSMC-Controlled Buck System

In the phase plane, the transient behavior of buck systems with multi-source disturbances and sensor-driver dynamics is described well by the PT. And it is crucial that we can further judge the response time of the arbitrary system state by the arbitrary PT. As mentioned above, the response time is composed of two parts, i.e., it consists of the sliding time *t_s_* and the reaching time *t_r_*. The reaching motion is not entirely controlled by the designed INTSMC controller and is also determined by its original state. Therefore, in the following, we will focus on PT analysis associated with different original points of the system, and the dynamic characteristic of the buck system under the control of INTSMC will be obtained later by further analysis with the system PT.

### 4.1. Existence of the Critical Surface

For the INTSMC-controlled buck converter in Figure 1a, by substituting (24)–(26) into (7), the closed-loop control system can be described as:

(53) $$\{\begin{array}{l} {\dot{x}}_{1}=x_{2}\\ {\dot{x}}_{2}=-\frac{1}{\lambda_{1}\gamma }x_{2}^{*2-\gamma }-\frac{\lambda_{2}}{\lambda_{1}\gamma }{x_{1}}^{*}x_{2}^{*1-\gamma }-D_{3}M\mathrm{sgn}\left(s\right)-\varepsilon_{u}\left(\mu_{u}\right)\\ {\dot{x}}_{3}=x_{1} \end{array}$$

From (53), it is not hard to see that the dynamic behavior of the system is affected by different initial points (*x*_1_(0), *x*_2_(0)). Therefore, it can be described by the PT in different initial states. In order to describe the PT of the buck system conveniently, in this paper, the concept of critical surface (CS) is introduced in the following.

Here, we can define a critical surface as *f_CS_*(***x***). For the reaching motion in the phase plane (*x*_1_, *x*_2_), it refers to the moment when the system reaches the sliding surface *s* = 0 from the initial point (*x*_1_(0), *x*_2_(0)). In order to define the critical surface *f_CS_*(***x***), it is reasonable to make a sufficiently small initial point in the *ε_s_*(*μ_x_*_1_, *μ_x_*_2_)-region around the ending point (0,0) as the initial point, despite moving in the direction departing from the origin. Therefore, the *f_CS_*_1_(***x***) in the region *s* > 0 with initial states (*x*_1_(0), *x*_2_(0)) ∈ *ε_s_*(*μ_x_*_1_, *μ_x_*_2_) can be derived as

(54) $$f_{CS1}\left(x\right):\{\begin{array}{l} {\dot{x}}_{1}=-x_{2}\\ {\dot{x}}_{2}=\frac{1}{\lambda_{1}\gamma }x_{2}^{*2-\gamma }+\frac{\lambda_{2}}{\lambda_{1}\gamma }{x_{1}}^{*}x_{2}^{*1-\gamma }+D_{3}M\mathrm{sgn}\left(s\right)+\varepsilon_{u}\left(\mu_{u}\right) \end{array}$$

Similarly, the *f_CS_*_2_(***x***) in the region *s* < 0 can also be derived as

(55) $$f_{CS2}\left(x\right):\{\begin{array}{l} {\dot{x}}_{1}=-x_{2}\\ {\dot{x}}_{2}=\frac{1}{\lambda_{1}\gamma }x_{2}^{*2-\gamma }+\frac{\lambda_{2}}{\lambda_{1}\gamma }{x_{1}}^{*}x_{2}^{*1-\gamma }-D_{3}M\mathrm{sgn}\left(s\right)+\varepsilon_{u}\left(\mu_{u}\right) \end{array}$$

According to the definitions of (54) and (55), PTs of the system’s dynamic behavior in (52) will finally reach the origin along *f_CS_*(***x***).

**Remark** **8.** *According to the definitions of (54) and (55), the trajectories of system (52) with the initial states on the CS will reach the origin along the CS.*

**Figure 8:**
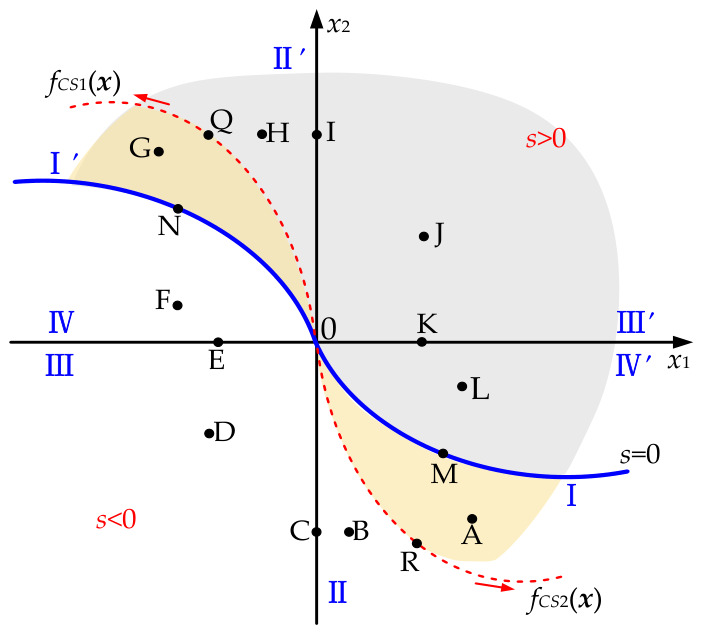
Diagram of the possible initial points and critical surfaces.

Combining (54) and (55), all possible initial points can be illustrated in Figure 8; they are sixteen symmetrical points about the origin and are divided into eight different groups. For clear presentation, we highlight these two critical surfaces, i.e., *f_CS_*_1_(***x***) and *f_CS_*_2_(***x***). And the whole phase plane is symmetrically divided into two regions by the nonlinear sliding mode surface *s* = 0. In each region, there are two different areas partitioned by the critical surface *f_CS_*(*x*), as shown in Figure 8: areas I, II, III and IV for the region *s* < 0, and areas I′, II′, III′ and IV′ for the region *s* > 0. Obviously, areas I and I′ are symmetrical according to the origin, as others.

Seen from (52), since there is sgn(*s*), considering that different values of the sliding variable *s* correspond to different states of the system with different initial points, here the PTs from different initial points to the original are discussed in the following three cases, respectively: *s* < 0, *s* > 0, and *s* = 0.

(1) **Case 1: *s* < 0**

In the case of *s* < 0, the possible initial points include A, B, C, D, E, F, and R, given in Figure 8. And the dynamic behavior of the INTSMC system associated with the CS is stated in the following Theorem 2.

**Theorem** **2.** *For the points A and B contained in the region s < 0, the PT from any initial point cannot cross f_CS1_(x_1_, x_2_), and only moves in a unique direction, while the PT at the initial point A close to s = 0 is the shortest one converging to the equilibrium origin.*

**Proof** **of Theorem 2.** For point A, since it is satisfied with ${\dot{x}}_{1}$ = x_2_ < 0, ${\dot{x}}_{2}$ > 0, the PT will move along the negative direction of x_1_ and the positive direction of x_2_; this means that over time, x_1_ will decrease, whereas x_2_ will increase, which could lead the trajectory to reach the SMC surface with x_1_ > 0 and x_2_ < 0 in Figure 8. Taking it into the system dynamic behavior in (52), the results indicate that the motion direction of the trajectory is only determined by its initial point.

We continue to prove the PT of point A cannot be through the critical surface *f_CS_*_1_(*x*_1_, *x*_2_). First, let the coordinates of point A be (*x*_1A_, *x*_2A_), and assume there is another point A′(*x*_1A′_, *x*_2A′_) located in the small vicinity *ε_H_*(*μ_x_*_1_, *μ_x_*_2_, *μ_u_*) of point A. According to the Lipschitz condition, there is

(56) $$\left\|F\left(A\right)-F\left(A^{\prime }\right)\right\|\leq k\left\|A-A^{\prime }\right\|$$

(57) $$F\left(x\right)=\left(x_{2},-\frac{1}{\lambda_{1}\gamma }x_{2}^{*2-\gamma }-\frac{\lambda_{2}}{\lambda_{1}\gamma }{x_{1}}^{*}x_{2}^{*1-\gamma }-D_{3}M\mathrm{sgn}\left(s\right)-\varepsilon_{u}\left(\mu_{u}\right)\right)$$

where *k* > 0 is a Lipschitz constant, and the function *F*(***x***) satisfies the differential equation in (52) according to (22). To further analyze the Lipschitz condition in (56), here defining *ζ* = ||*F*(A) − *F*(A′)||, (57) can be further expressed as

(58) $$\begin{array}{cc} \left\|F\left(A\right)-F\left(A^{\prime }\right)\right\| & ={\left[{\left(x_{1A}-x_{1A^{\prime }}\right)}^{2}+\frac{1}{{\lambda_{1}}^{2}\gamma^{2}}{\left(-{x_{2A}^{*}}^{2-\gamma }-\lambda_{2}{x_{1A}}^{*}{x_{2A}^{*}}^{1-\gamma }+{x_{2A^{\prime }}^{*}}^{2-\gamma }+\lambda_{2}{x_{1A^{\prime }}}^{*}{x_{2A^{\prime }}^{*}}^{1-\gamma }\right)}^{2}\right]}^{\frac{1}{2}}\\ & ={\left[{\left(x_{1A}-x_{1A^{\prime }}\right)}^{2}+\frac{1}{{\lambda_{1}}^{2}\gamma^{2}}{\left(\varphi \left(A\right)-\varphi \left(A^{\prime }\right)\right)}^{2}\right]}^{\frac{1}{2}} \end{array}$$

In the region [A, A′], the function *φ*(*ξ*) is differentiable for *ξ* ∈ [A, A′], *ξ* = [*ξ*_A_, *ξ*_A_′], such that the Rolle mean-value theorem can be applied, which means

(59) $${\left\|\varphi \left(A\right)-\varphi \left(A^{\prime }\right)\right\|}^{2}={\left\|\nabla \varphi \left(\xi \right)\left(A-A^{\prime }\right)\right\|}^{2}\leq {\left\|\nabla \varphi \left(\xi \right)\right\|}^{2}{\left\|\left(A-A^{\prime }\right)\right\|}^{2}$$

Therefore, we have the following inequality:

(60) $$\begin{array}{cc} \left\|F\left(A\right)-F\left(A^{\prime }\right)\right\| & \leq {\left[{\left(x_{1A}-x_{1A^{\prime }}\right)}^{2}+\frac{1}{{\lambda_{1}}^{2}\gamma^{2}}{\left\|\nabla \varphi \left(\xi \right)\right\|}^{2}{\left\|A-A^{\prime }\right\|}^{2}\right]}^{\frac{1}{2}}\\ & \leq {\left[A-{A^{\prime }}^{2}+\frac{1}{{\lambda_{1}}^{2}\gamma^{2}}{\left\|\nabla \varphi \left(\xi \right)\right\|}^{2}{\left\|A-A^{\prime }\right\|}^{2}\right]}^{\frac{1}{2}}\\ & ={\left(1+\frac{1}{{\lambda_{1}}^{2}\gamma^{2}}{\left\|\nabla \varphi \left(\xi \right)\right\|}^{2}\right)}^{\frac{1}{2}}\left\|A-A^{\prime }\right\| \end{array}$$

The Lipschitz condition holds for the function *F*(***x***) in the region [A, A′] with a Lipschitz constant *L* = min*_ξ_*_∈[A, A′]_[1 + 1/(*λγ*)^2^||*φ*(*ξ*)||^2^]^1/2^. Based on the above analysis, it suggests that the Lipschitz condition is established completely in *ε_H_*(*μ_x_*_1_, *μ_x_*_2_, *μ_u_*) of point A, indicating the solution of the differential (52) is unique over time in *s* < 0.

**Figure 9:**
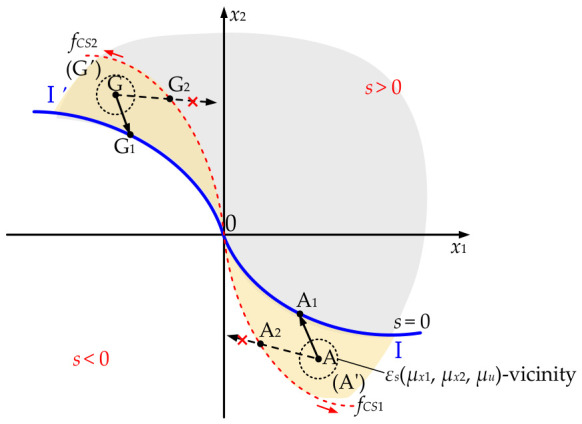
Diagram of the trajectory analysis of point A in the phase plane.

According to (52), it can be obtained that ${\dot{x}}_{1}$ < 0, which means that any point on the PT is always differentiable, and the slope of the PT is always negative in area I, such as the trajectory A → A_1_ in area I, shown in Figure 9. If there is another trajectory from point A that can cross the *f_CS_*_1_(*x*_1_, *x*_2_) at the point A_2_, then, according to *Remark 7*, the trajectory will move to the origin along the CS. The point A_2_ on this trajectory is not differentiable, which means the trajectory cannot cross the CS for any initial states in area I. The above deduction can be similarly obtained for the region *s* > 0 (such as point G in Figure 9).

Similarly, for point B, due to ${\dot{x}}_{1}$ < 0, ${\dot{x}}_{2}$ > 0, the PT will move along the negative direction of *x*_1_ and along the positive direction of *x*_2_, respectively; this also means that over time *x*_1_ will decrease, whereas *x*_2_ will increase, as in the case of A. However, the PT starting from B cannot cross *f_CS_*_1_(*x*_1_, *x*_2_); it will pass through the negative axis of *x*_1_ and the negative axis of *x*_2_, respectively, and finally reach the origin along *s* = 0 in area IV. This completes the proof of Theorem 2. □

**Figure 10:**
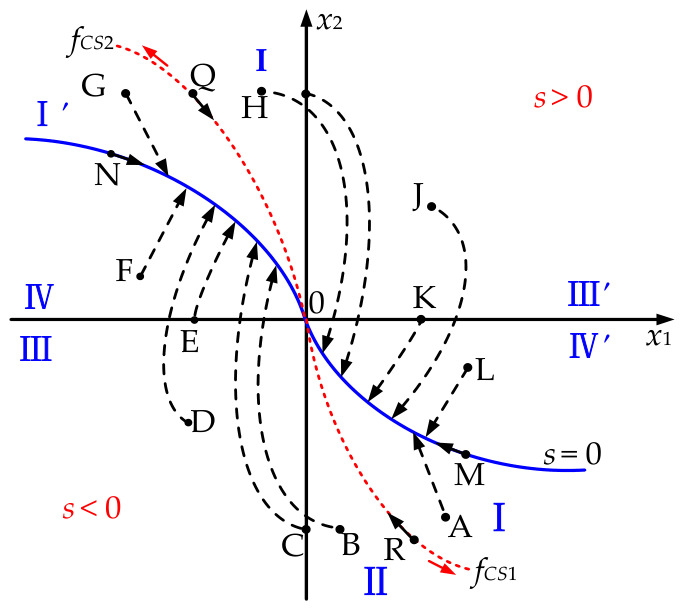
Diagram of trajectories of the possible initial points.

Based on the above trajectory analysis of points A and B, the trajectories of other possible initial points, i.e., C, D, E, F, and R, located in Figure 8, can be illustrated in Figure 10. Specifically, the point R lying on *f_CS_*_1_(*x*_1_, *x*_2_) will reach the origin along *f_CS_*_1_(*x*_1_, *x*_2_), which has been mentioned in Remark 7 above.

(2) **Case 2: *s* > 0**

In this case, the possible initial points include G, Q, H, I, J, K, and L, given in Figure 8. Point G is symmetric with A about the origin in area I′ and satisfies Theorem 2, while point H is symmetric with B about the origin in area II′, holding similar dynamic behavior to H. The convergence trajectory of other state points is shown in Figure 10.

(3) **Case 3: *s* = 0**

In this case of *s* = 0, which is the SM surface of INTSMC, actually, the possible initial points contain *M* and *N*, as shown in Figure 8. Due to the constraint of the equivalent control law *u_eq_* of the INTSMC in (24), they finally converge to the origin along *s* = 0.

### 4.2. Response Time Estimation of the INTSMC-Controlled Buck System

According to the dynamic movement of the INTSMC system described in Figure 5, the estimation algorithm of response time *t_c_* of the system is conveniently closely related to the reaching time *t_r_* and sliding time *t_s_*, and satisfies *t_c_* = *t_r_* + *t_s_*.

For the sliding time *t_s_* associated with *s* = 0, which can be obtained from (23):

(61) $$t_{s}=\frac{{\left|x_{2}^{\gamma -1*}\left(t_{r}\right)\right|}^{-1}\left|\frac{{\left[{x_{1}}^{*}\left(0\right)+{x_{3}}^{*}\left(0\right)\right]}^{-1}+\varepsilon_{s}\left(\mu_{x1},\mu_{x2},\mu_{u}\right)}{{\left[{x_{2}}^{*}\left(t_{r}\right)\right]}^{-1}}+1\right|}{\left|{\left[\frac{{x_{1}}^{*}\left(0\right)}{{x_{2}}^{*}\left(t_{r}\right)}\right]}^{-1}+\lambda_{2}\right|}$$

**Figure 11:**
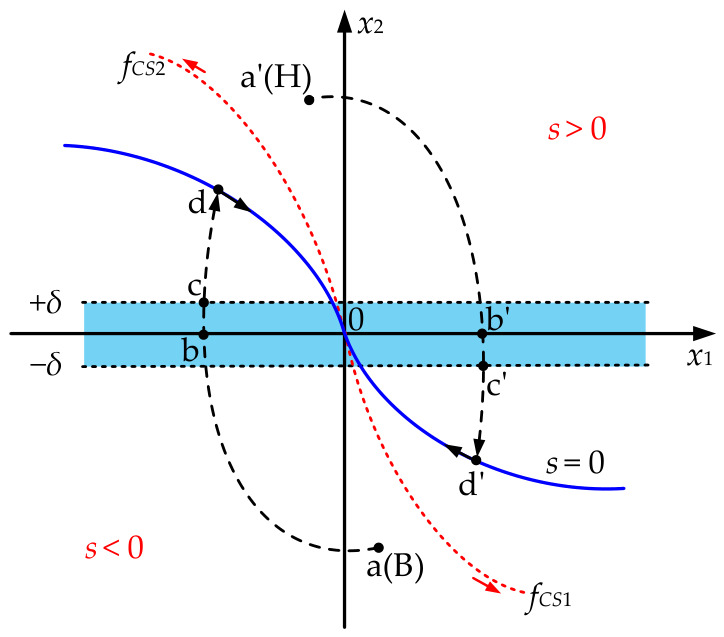
Diagram of response time estimation of points B and H.

We consider the region of *s* < 0. For the reaching time *t_r_*, we give full consideration to the influence of the initial positions of system states. In this paper, we take the initial point B(*x*_1B_(0), *x*_2B_(0)) in the case of *s* < 0 as an example to calculate it piece by piece based on the CS *f_CS_*_1_(*x*_1_, *x*_2_) and the trajectories of the possible initial points in Figure 11. In detail, the *t_r_* is composed of three parts, i.e., a → b → c → d. Taking into account the PWM used in Figure 1, here we can design the hysteresis band variable as *δ* > 0, shown in Figure 11 (also mentioned in Figure 5), where +*δ* and −*δ* are used to facilitate the analysis of initial points crossing the *x*_1_ axis.

(1) Point a → b(*t*_ab_): since the initial state *x*_2B_(0) < 0 in Figure 11, ${\dot{x}}_{2}$ ≥ *D*_3_*M* can be obtained from (52), so that the time *t*_ab_ can be calculated as
  (62) $$t_{ab}\leq \frac{x_{2B}(0)}{D_{3}M}\leq \frac{x_{2B}(0)}{M\psi_{D3\max}}$$

(2) Point b → c(*t*_bc_): Since −*δ* ≤ *x*_2_ < 0, there certainly exists a *k* in (56) satisfying 0 < *k* < 1 and ${\dot{x}}_{2}$ ≥ *kD*_3_*M*. Therefore, *t*_bc_ can be estimated as

(63) $$t_{\mathrm{bc}}\leq \frac{\delta }{kM\psi_{D3\max}}$$

At the same time, taking the inequality ${\dot{x}}_{2}$ ≥ *kMψ_D_*_3max_ into (52), we can get

(64) $$x_{2}^{*2-\gamma }+{x_{1}}^{*}x_{2}^{*1-\gamma }\leq \lambda_{1}\gamma M\psi_{D3\max}\left(1-k\right)+\varepsilon_{u}\left(\mu_{u}\right)$$

Substituting *x*_2_ = *δ* into (64), then *δ* can be expressed in terms of *k* such that

(65) $$\delta \leq {\left[\lambda_{1}\gamma M\psi_{D3\max}\left(1-k\right)-X_{2}^{1-\gamma }{x_{2B}}^{-1}\left(0\right)\right]}^{\gamma -2}e^{\left(2-\gamma \right)t}+\varepsilon_{u}\left(\mu_{u}\right)$$

(3) Point c → d(*t*_cd_): Consider the motion process from point c to d, which is satisfied with *x*_2_ > *δ* > 0. Since *x*_2_*^γ^^−1^ > 0, combining with the Lyapunov function in (33), there is *η*[*x*_2_*(*t*_ac_)] < *η*[*x*_2_*(*t*_cd_)] within the motion process c → d in Figure 11. Therefore, (33) can be rewritten as
  (66) $$\dot{V}\leq -\eta \left[{x_{2}}^{*}(t_{\mathrm{cd}})\right]V^{\frac{1}{2}}$$

The time *t*_cd_ can be further obtained from (66) as

(67) $$t_{\mathrm{cd}}\leq \frac{2V^{\frac{1}{2}}\left(t_{\mathrm{cd}}\right)}{\eta \left[{x_{2}}^{*}\left(t_{\mathrm{cd}}\right)\right]}=\frac{2V^{\frac{1}{2}}\left(t_{\mathrm{cd}}\right)}{M\lambda_{1}\gamma {x_{2}}^{*\gamma -1}\left(t_{\mathrm{cd}}\right)}\;\leq \frac{2V^{\frac{1}{2}}\left(t_{\mathrm{cd}}\right)}{M\lambda_{1}\gamma \delta^{\gamma -1}}$$

At time *t*_cd_, there is

(68) $$\begin{array}{cc} V^{\frac{1}{2}}(t_{\mathrm{cd}}) & =\frac{|s(t_{\mathrm{cd}})|}{\sqrt{2}}\\ & =\frac{\sqrt{2}}{2}\left|{x_{1}}^{*}(t_{\mathrm{cd}})+\lambda_{1}x_{2}^{*\gamma }(t_{\mathrm{cd}})+\lambda_{2}{x_{3}}^{*}(t_{\mathrm{cd}})\right|\\ & \leq \frac{\sqrt{2}}{2}\left|x_{1}(t_{\mathrm{bd}})+\lambda_{1}\delta^{\gamma }+\lambda_{2}\int_{0}^{t_{\mathrm{bd}}}x_{1}dt+\varepsilon_{s}\left(\mu_{x1},\mu_{x2},\mu_{u}\right)\right| \end{array}$$

Hence, the analysis of *x*_1_*(*t*_cd_) is replaced by *x*_1_*(*t*_bd_). Since there is *x*_2_ = 0 at *t*_b_, from the system dynamics in (52), we can get

(69) $${\dot{x}}_{2}={\ddot{x}}_{1}=D_{3}M-\varepsilon_{u}\left(\mu_{u}\right)\leq M\psi_{D3\max}$$

Focusing on *x*_2_, *t*_bd_ can be further obtained from (67) as

(70) $$t_{\mathrm{bd}}\geq \frac{x_{2B}\left(0\right)}{M\psi_{D3\max}}$$

Focusing on *x*_1_, by selecting the appropriate parameter *ψ*_2_ with *X*_2_ ≥ *ψ_x_*_2_ ≥ |*x*_2B_(0)| in (67), it yields

(71) $$\begin{array}{cc} \left|x_{1}(t_{\mathrm{bd}})\right| & =x_{1}(0)+\frac{1}{2}\left|D_{3}M-\varepsilon_{u}\left(\mu_{u}\right)\right|{t_{\mathrm{bd}}}^{2}+x_{2B}(0)t_{\mathrm{bd}}\\ & \leq x_{1}(0)+\frac{1}{2}M\psi_{D3\max}{t_{\mathrm{bd}}}^{2}+\psi_{x2}t_{\mathrm{bd}} \end{array}$$

Therefore, by substituting (68), (69), and (71) into (67), *t*_cd_ can be rewritten as

(72) $$\begin{array}{cc} t_{\mathrm{cd}} & \leq \frac{\sqrt{2}\left|x_{1}(0)+\frac{1}{2}\frac{{x_{2B}}^{2}\left(0\right)}{M\psi_{D3\max}}+\psi_{x2}\frac{x_{2B}\left(0\right)}{M\psi_{D3\max}}+\lambda_{1}\delta^{\gamma }+\lambda_{2}\int_{0}^{t_{\mathrm{bd}}}x_{1}dt+\varepsilon_{s}\left(\mu_{x1},\mu_{x2},\mu_{u}\right)\right|}{M\lambda_{1}\gamma \delta^{\gamma -1}\left(t_{\mathrm{cd}}\right)}\;\\ & \leq \frac{\sqrt{2}\left|x_{1}(0)+\frac{3}{2}\frac{{\psi_{x2}}^{2}}{M\psi_{D3\max}}+\lambda_{1}\delta^{\gamma }+\lambda_{2}\int_{0}^{t_{\mathrm{bd}}}x_{1}dt+\varepsilon_{s}\left(\mu_{x1},\mu_{x2},\mu_{u}\right)\right|}{M\lambda_{1}\gamma \delta^{\gamma -1}\left(t_{\mathrm{cd}}\right)} \end{array}$$

From the position of d, it can be seen from Figure 11 that *V*(*t*_d_) = 0, which indicates that the system state approaches zero gradually from a certain value along with the rate in (66).

Therefore, the reaching time *t_r_* = *t*_ab_ + *t*_bc_ + *t*_cd_ can be obtained by combining (71) as

(73) $$\begin{array}{cc} t_{r} & \leq t_{\mathrm{ab}}+t_{\mathrm{bc}}+t_{\mathrm{cd}}\\ & \leq \frac{x_{2B}(0)}{M\psi_{D3\max}}+\frac{\delta }{kM\psi_{D3\max}}+\frac{\sqrt{2}\left|x_{1}(0)+\frac{3}{2}\frac{{\psi_{x2}}^{2}}{M\psi_{D3\max}}+\lambda_{1}\delta^{\gamma }+\lambda_{2}\int_{0}^{t_{\mathrm{bd}}}x_{1}dt+\varepsilon_{s}\left(\mu_{x1},\mu_{x2},\mu_{u}\right)\right|}{M\lambda_{1}\gamma \delta^{\gamma^{-1}}\left(t_{\mathrm{cd}}\right)} \end{array}$$

Obviously, (73) can also be applied to points C, D, and E, which need to cross the *x*_1_ and *x*_2_ axes in Figure 10. Specifically, considering that the trajectories of points A and F do not need to cross the axis, *t_r_* can be calculated in the same way. According to (33), it can be obtained as

(74) $$t_{r}\leq \frac{\sqrt{2}\left|x_{1}(0)+\lambda_{1}\delta^{\gamma }+\lambda_{2}\int_{0}^{t_{\mathrm{bd}}}x_{1}dt+\varepsilon_{s}\left(\mu_{x1},\mu_{x2},\mu_{u}\right)\right|}{{M\lambda_{1}\gamma x_{2}}^{*\gamma -1}\left(0\right)}$$

For the region of *s* > 0, here we select the point H shown in Figure 10, whose trajectory of the system is symmetric about the origin with point B according to (52); therefore, the estimation expressions of *t_r_* can be referred to in (73), the same as points I, J and K, while points G and L are symmetric about the origin with A and F, which can be referred to in (74).

### 4.3. Practical Design Guideline for Parameter Selection

**Figure 12:**
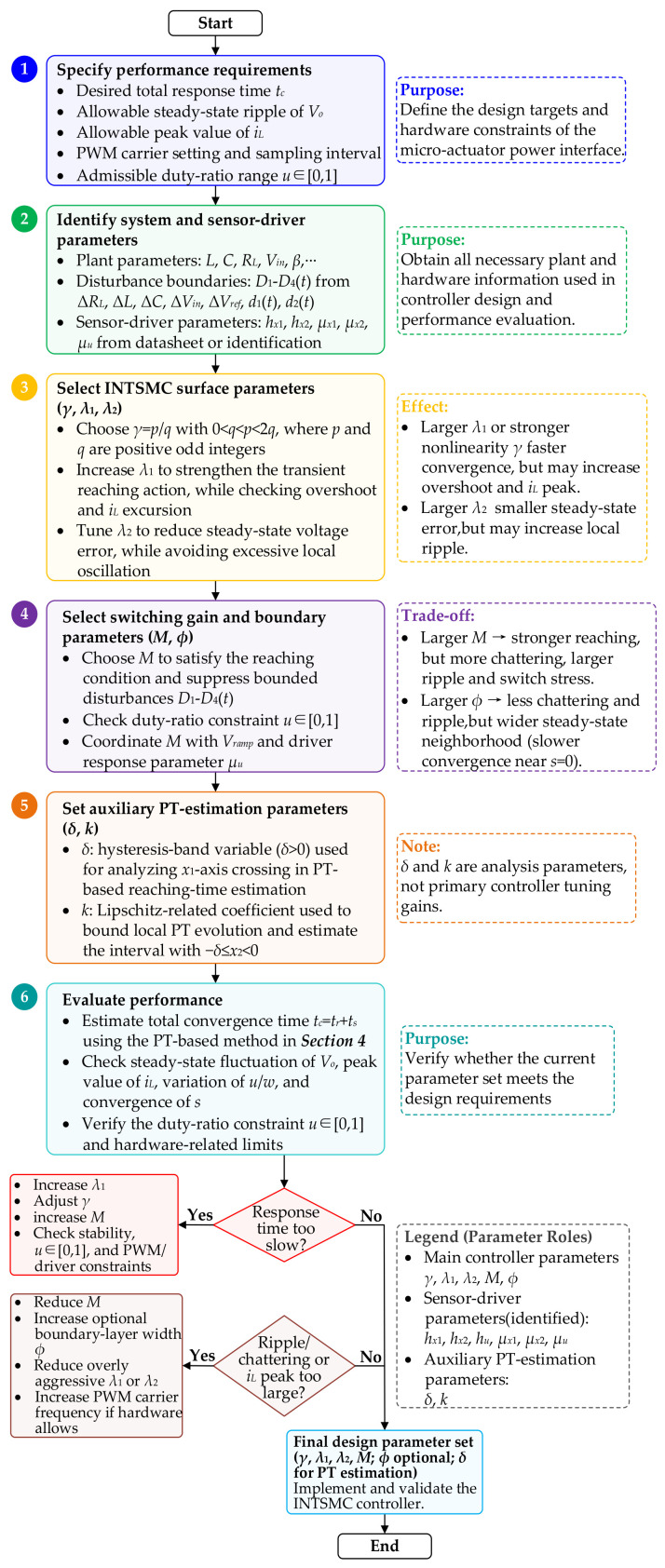
Practical parameter-selection procedure for the INTSMC-controlled buck converter.

To improve the engineering readability of the PT-based response-time estimation, a practical parameter-selection procedure is summarized in Figure 12.

The parameters involved in Section 3 and Section 4 can be divided into three groups. The first group contains the main INTSMC tuning parameters, including *γ*, λ_1_, λ_2_, and *M*, which directly affect the sliding surface, reaching behavior, steady-state voltage error, and switching intensity. The second group contains the sensor-driver parameters, including *h_x_*_1_, *h_x_*_2_, *h_u_*, *μ_x_*_1_, *μ_x_*_2_, and *μ_u_*, which are obtained from datasheets or model identification rather than manually tuned as controller gains. The third group contains auxiliary PT-estimation parameters, such as *δ* and *k*. Here, *δ* is used as a hysteresis-band variable in the reaching-time estimation, while *k* is the Lipschitz constant used for local PT uniqueness analysis. Therefore, *δ* and *k* should be regarded as analysis parameters rather than primary control parameters.

As shown in Figure 12, the design starts from the required response time, allowable steady-state fluctuation of *V_o_*, allowable peak value of *i_L_*, and PWM/duty-ratio constraints. After identifying the plant parameters, disturbance boundaries *D*_1_ to *D*_4_(*t*), and sensor-driver parameters, *γ*, *λ*_1_, and *λ*_2_ are selected to shape the sliding surface. Increasing *λ*_1_ or *M* usually strengthens the reaching action and shortens the transient process, but may increase *i_L_* excursion, residual chattering, and *V_o_* fluctuation. Increasing *λ*_2_ helps reduce the steady-state voltage error, but an excessively large value may introduce local oscillation near *s* = 0. Therefore, after estimating *t_c_* = *t_r_* + *t_s_*, the obtained parameter set should be checked using *V_o_* fluctuation, *i_L_* peak, variation of *u*/*w*, and convergence of *s*. If the response is too slow, *λ*_1_, *γ*, or *M* can be adjusted within the stability, *u* ∈ [0, 1], and PWM/driver constraints. If the ripple or chattering is too large, *M* should be reduced, the optional boundary-layer width *ϕ* can be increased, or the sliding-surface parameters should be softened.

## 5. Simulation Analysis and Verification

To validate the proposed INTSMC controller with multi-source disturbances and sensor-driver dynamics in Figure 6, the parameters of the buck converter are listed in Table 1, which can guarantee that the buck converter will work in CCM [31]. The variable divider resistor *R*_2_ is fixed at 3 kΩ so that the ratio *β* = *R*_1_/(*R*_1_ + *R*_2_) = 0.25, and the variable resistor *R_m_* for providing the voltage difference can be further fixed at 5 kΩ. All simulations were carried out in MATLAB/Simulink R2018b using a fixed-step ode3 (Bogacki-Shampine) solver. The fixed-step size was *T_step_* = 1 × 10^−5^ s. Since the PWM switching frequency is *fs* = 10 kHz, the corresponding switching period is *T_sw_* = 1 × 10^−4^ s, so the selected step size is *T_sw_*/10. This setting provides sufficient numerical resolution for the fixed-frequency PWM response and sensor-driver dynamic effects.

The simulation cases are designed to emulate local voltage regulation requirements in compact actuator-driven microsystems. In such applications, the buck converter may experience source-side voltage variation, actuator-related load change, passive component tolerance, and non-ideal sensor-driver dynamics within a compact hardware layout. Therefore, the simulations focus on voltage recovery, current boundedness, PWM-compatible control action, and PT convergence under these operating conditions.

These parameters are selected to represent a low-voltage local power interface rather than a high-power conversion stage, which is consistent with the voltage regulation level required by compact actuator-related electronic modules.

**Remark** **9.** *The selected L = 1 mH and C = 3.2 mF are conservative benchmark parameters for control-oriented validation, rather than minimum-size components for a high-frequency miniaturized converter. Under V_in_ = 16 V, V_o_ = 8 V, R_L_ = 10 Ω, and f_s_ = 10 kHz, the nominal load current is 0.8 A and the duty ratio is 0.5. The estimated inductor-current ripple is Δi_L_ = (V_in_ − V_o_)D/(Lf_s_) ≈ 0.4 A, so the minimum inductor current remains positive and CCM is maintained. The critical inductance is L_crit_ = (1 − D)R_L_/(2f_s_) = 0.25 mH, lower than the selected 1 mH. Meanwhile, C = 3.2 mF gives an estimated capacitive voltage ripple of ΔV_o_ ≈ Δi_L_/(8Cf_s_) ≈ 1.56 mV, providing a stiff local voltage buffer for the actuator-side load. Smaller L and C values can be used in high-frequency designs after retuning the controller parameters.*

**Remark** **10.** *The nominal simulation adopts the averaged buck converter model with ideal L and C values. Component parasitics, such as inductor winding resistance and capacitor equivalent series resistance (ESR), are not modeled as separate circuit elements in the nominal simulation. Instead, their effects are regarded as bounded non-ideal perturbations and are covered by the multi-source disturbance terms in (6)–(10). In the hardware experiment, these parasitic effects are inherently included in the real inductor, capacitor, printed circuit board (PCB) traces, power switch, sensor links, and driver circuit.*

### 5.1. Sensor-Driver Dynamic Model Settings

**Figure 13:**
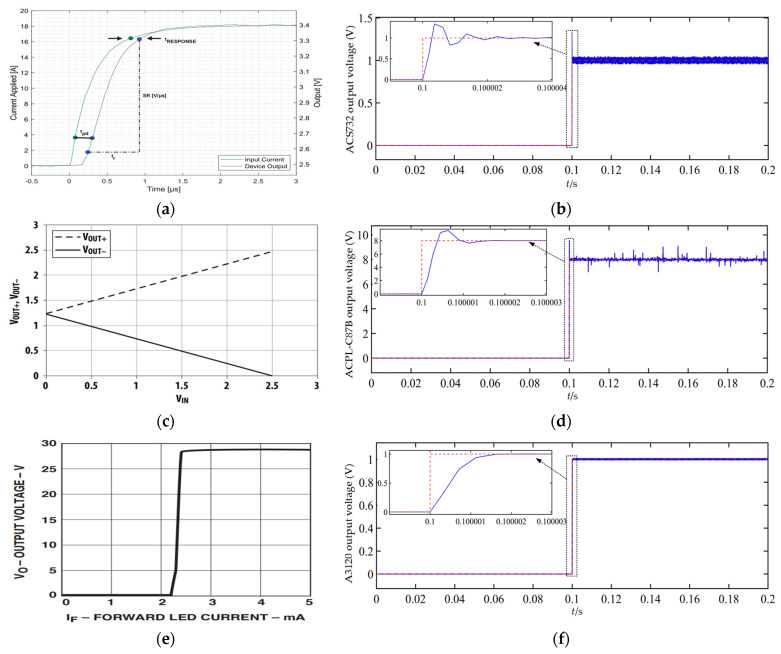
Model identification and evaluation of sensors and the driver: (**a**) input–output characteristic of the Hall current sensor ACS732 [32]; (**b**) the step response of the identified model of the ACS732; (**c**) input–output characteristic of the Hall voltage sensor ACPL-C87B [33]; (**d**) the step response of the identified model of the ACPL-C87B; (**e**) input–output characteristic of the isolated driver A3120 [34]; (**f**) the step response of the identified model of the A3120.

For the Hall current sensor ACS732, Hall voltage sensor ACPL-C87B, and the isolated driver A3120 in the buck converter system in Figure 2, the test data in their chip manual [32,33,34] are shown in Figure 13a,c,d. The selected sensor and driver devices are used here as representative chip-level links in compact converter control loops, where their response time and static gain directly affect the measured feedback variables and the effective switching action.

By using the software (MATLAB 2018a) of data recognition tools with engauge and the linear fitting tool with MATLAB 2018a Identification Toolbox, their transfer functions can be further described, as shown in Table 2. The step response of identification models in Table 2 is illustrated in Figure 13b,d,f, and the stable condition of the Hall sensors and driver in (14), (18), and (19) can be further satisfied.

### 5.2. Control Parameter Sensitivity Analysis

**Figure 14:**
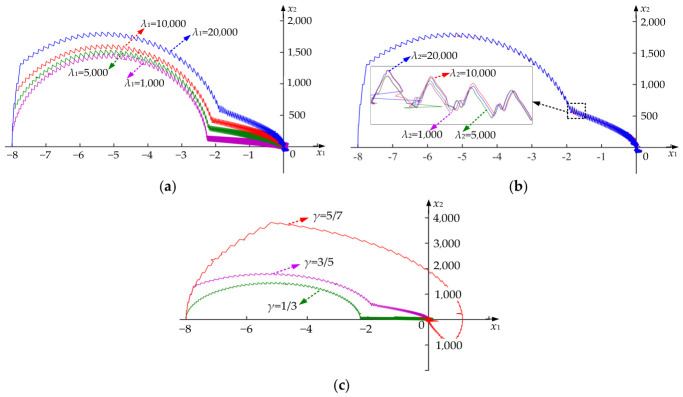
INTSMC parameter sensitivity analysis: (**a**) controller parameter *λ*_1_; (**b**) controller parameter *λ*_2_; (**c**) controller parameter *γ*.

To clarify the influence of the main control parameters on the transient behavior, a phase-plane sensitivity analysis is carried out. In this test, only one parameter is changed at a time, while the other parameters and the initial operating condition are kept unchanged. The investigated parameters include the linear sliding coefficient *λ*_1_, the nonlinear sliding coefficient *λ*_2_, and the fractional power exponent *γ*. The corresponding PTs are shown in Figure 14a–c, where *x*_1_ and *x*_2_ denote the voltage tracking error and its derivative, respectively.

As shown in Figure 14a, increasing *λ*_1_ enlarges the transient excursion of the PT. When *λ*_1_ is set to 10,000 or 20,000, the trajectory moves through a wider region, and the peak value of *x*_2_ becomes larger, indicating a more aggressive transient correction. In contrast, *λ*_1_ = 1000 gives a more compact trajectory with a smaller velocity excursion, which helps avoid excessive control action and dynamic fluctuation.

Figure 14b compares the influence of *λ*_2_. It can be observed that *λ*_2_ has a weaker effect on the global PT than *λ*_1_, while its influence is more evident in the local oscillatory region near the approaching trajectory. A larger *λ*_2_ slightly enhances the nonlinear correction, but it may also increase local fluctuation. Therefore, *λ*_2_ = 1000 is selected to balance convergence behavior and control smoothness.

The effect of *γ* is shown in Figure 14c. A smaller exponent, such as *γ* = 1/3, reduces the peak value of *x*_2_, but the trajectory stays near the *x*_1_-axis for a longer distance, which may slow down the final reaching process. When *γ* = 5/7, the convergence action becomes stronger, but the trajectory shows a much larger excursion and obvious overshoot near the origin. By comparison, *γ* = 3/5 provides a more balanced response between convergence speed and transient amplitude. Based on the above observations, the control parameters are chosen as *λ*_1_ = 1000, *λ*_2_ = 1000, and *γ* = 3/5 in the simulations. In (25), the switching gain *M* can be set to 1.2 to avoid the introduction of large chattering after considering converter sensor-driver dynamics and multi-source disturbances.

### 5.3. Buck Converter Sensor-Drive Dynamic Analysis

**Figure 15:**
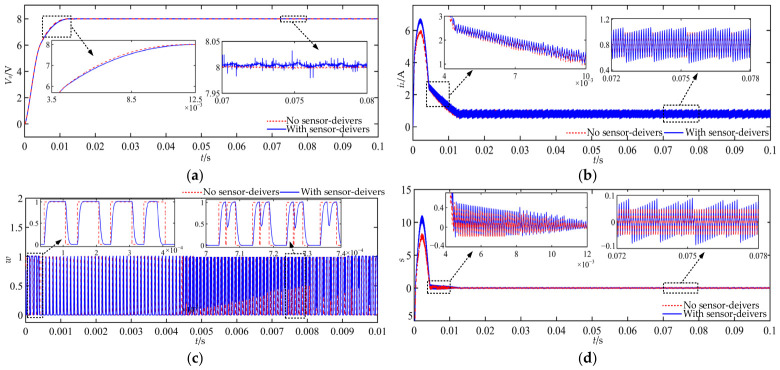
Comparison of the INTSMC-controlled buck converter with or without sensor-drivers: (**a**) output voltage *V*_o_; (**b**) inductor current *i*_L_; (**c**) control law *w*; (**d**) sliding variable *s*.

Figure 15 compares the dynamic responses of the INTSMC-controlled buck converter with and without sensor-driver dynamics. The output voltage responses in Figure 15a show that both cases can track the reference value of 8 V within a short transient process. Compared with the ideal case without sensor-driver dynamics, the response with sensor-driver dynamics presents a slight transient delay and small steady-state fluctuation, but the voltage regulation accuracy is still maintained.

As shown in Figure 15a, the output voltage in both cases can rapidly converge to the reference value of 8 V. Compared with the ideal case without sensor-driver dynamics, the response with sensor-driver dynamics shows a slightly slower rising process in the enlarged transient region. In the steady-state enlarged view, small voltage fluctuations can also be observed after considering the sensor-driver links. However, the fluctuation amplitude remains limited, and the output voltage is still regulated around the desired value, indicating that the voltage tracking accuracy is not significantly degraded.

Figure 15b presents the corresponding inductor current responses. During the startup transient, the case with sensor-driver dynamics has a higher current peak, which means that the non-ideal feedback and driving links slightly increase the transient current stress. In the steady-state region, the inductor current also exhibits more evident ripple than in the ideal case. This phenomenon is consistent with the practical influence of sensor and driver dynamics, since the feedback measurement and duty-cycle action are no longer perfectly synchronized with the ideal control command.

The control law shown in Figure 15c further explains the difference between the two cases. Without sensor-driver dynamics, the control signal follows the ideal switching command more directly. After introducing sensor-driver dynamics, the actual control signal presents a certain waveform delay and local distortion, as shown in the enlarged views. Even so, the control input remains within the admissible switching range, which means that the proposed control law can still be implemented in a PWM-compatible manner under non-ideal driving conditions.

Figure 15d shows the evolution of the sliding variable. In both cases, the sliding variable converges rapidly toward the neighborhood of zero after a short transient process. The case with sensor-driver dynamics produces a larger initial peak and stronger local oscillations during the reaching stage, but these oscillations are gradually suppressed as the system approaches steady state. Therefore, although the sensor-driver dynamics introduce additional transient fluctuation, the proposed INTSMC still maintains the reaching ability and robust regulation performance of the closed-loop buck converter.

**Figure 16:**
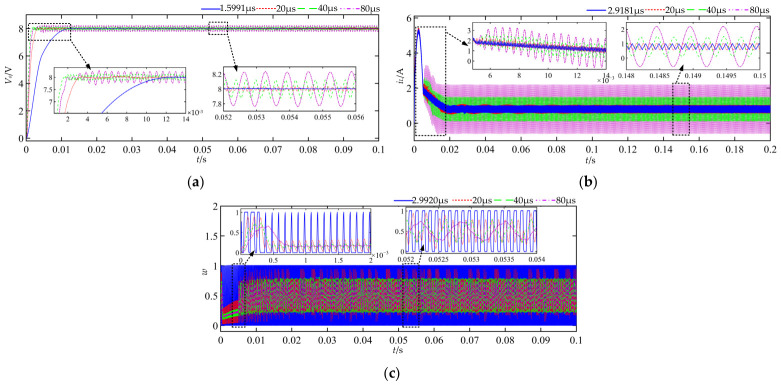
Parameter analysis of sensor-driver: (**a**) output voltage *V*_o_; (**b**) inductor current *i*_L_; (**c**) control law *w*.

To further investigate the influence of sensor-driver dynamic parameters, a parameter analysis is carried out by changing the equivalent response time of the sensor or driver link. In this test, the nominal response times obtained from the selected devices are compared with enlarged values of 20 μs, 40 μs, and 80 μs. The results in Figure 16a–c are used to evaluate how the non-ideal dynamic characteristics of the voltage sensor, current sensor, and driving link affect the voltage regulation, current response, and control action of the INTSMC-controlled buck converter.

Figure 16a shows the output voltage responses under different voltage-sensor dynamic parameters. When the nominal response time of 1.5991 μs is used, the output voltage rises more smoothly and finally remains close to the reference value with very small fluctuation. As the response time increases to 20 μs, 40 μs, and 80 μs, the voltage appears to approach the reference value faster in the early transient stage, but the steady-state ripple becomes more evident. This indicates that a slower sensing link may introduce feedback lag, which changes the equivalent closed-loop correction process and causes additional voltage oscillation around the reference value.

The influence of the current-sensor dynamic parameter is presented in Figure 16b. With the nominal response time of 2.9181 μs, the inductor current shows a relatively smooth transient decay and limited steady-state ripple. When the response time is increased, the current ripple becomes obviously larger, especially for the 80 μs case. This result suggests that the current feedback dynamic has a direct influence on the inductor current regulation. A slower current-sensing process weakens the synchronization between the actual current variation and the feedback signal used by the controller, thereby increasing the local current fluctuation.

Figure 16c compares the control law under different driver dynamic parameters. For the nominal response time of 2.9920 μs, the control signal can respond rapidly to the controller command and maintain a regular switching behavior. With the increase in the driver response time, the control signal presents more visible distortion and delayed action in the enlarged views. Although the control law remains within the admissible range, the slower driver dynamics reduce the accuracy of duty-cycle execution and may enlarge the ripple of the electrical states. Therefore, the nominal sensor-driver parameters are adopted in the subsequent simulations, since they provide a better balance between transient response, steady-state fluctuation, and PWM-compatible control action.

### 5.4. Performance Evaluation Under Multi-Source Disturbances and Sensor-Driver Dynamics

Table 3 summarizes the disturbance settings used in this case. The sensor-driver dynamics are considered throughout the whole simulation for both the disturbance-free and disturbed cases. The difference is that, in the disturbed case, circuit parameter perturbations, time-varying noise, input-voltage variations, and reference-voltage changes are successively introduced to examine the robustness of the proposed INTSMC.

In this setting, the load resistance variation is used to emulate actuator-related electrical load change, the reference-voltage steps represent different voltage regulation commands for actuator-side circuits, and the time-varying disturbances describe noise effects that may occur in densely integrated microsystem hardware.

**Figure 17:**
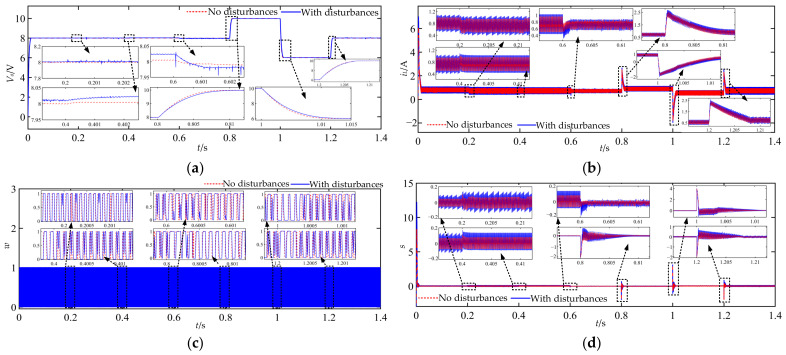
Comparison of the INTSMC-controlled buck converter with sensor-driver dynamics under multi-source disturbances: (**a**) output voltage *V*_o_; (**b**) inductor current *i*_L_; (**c**) control law *w*; (**d**) sliding variable *s*.

Based on the disturbance settings in Table 3, Figure 17 compares the performance of the INTSMC-controlled buck converter under sensor-driver dynamics with and without multi-source disturbances. This case is more complex than the previous single-factor tests because the parameter perturbations and noise disturbances are maintained after *t* = 0.2 s, while input-voltage changes and reference-voltage steps are further introduced at different time instants. Therefore, the simulation can be used to verify whether the proposed controller can maintain voltage regulation, current boundedness, PWM-compatible control action, and sliding convergence under coupled disturbances.

Figure 17a shows the *V_o_* response. Before *t* = 0.2 s, the *V_o_* rapidly reaches the initial *V_ref_* value of 8 V. After the parameter perturbations and noise disturbances are introduced, the disturbed case only shows a small local ripple around the reference voltage. When the *V_in_* changes at *t* = 0.4 s and *t* = 0.6 s, the *V_o_* presents limited deviation and then returns to the reference neighborhood. At *t* = 0.8 s, *t* = 1.0 s, and *t* = 1.2 s, the controller tracks the new *V_ref_* values of 10 V, 6 V, and 8 V, respectively. Although the disturbed case exhibits slightly larger fluctuation than the disturbance-free case, the overall tracking performance is still maintained.

The *i_L_* response is presented in Figure 17b. After *t* = 0.2 s, the *i_L_* ripple becomes more evident because the *L*, *C*, *R*, and *d*_1_(*t*), *d*_2_(*t*) are changed simultaneously. At the *V_in_* and *V_ref_* transition instants, the *i_L_* produces transient peaks or dips to rebalance the input and output power of the buck converter. The *i_L_* variation is particularly obvious at *t* = 0.8 s, *t* = 1.0 s, and *t* = 1.2 s because the *V_ref_* steps directly change the required voltage regulation level and output power demand. Nevertheless, the current remains bounded and gradually returns to a stable ripple range after each disturbance or reference change.

Figure 17c gives the corresponding control law *w*. Under the disturbed condition, the control signal *w* presents denser switching transitions than in the disturbance-free case, especially when *C*, *L*, *R*, *d*_1_(*t*), and *d*_2_(*t*) are changed at *t* = 0.2, and when *V_in_* and *V_ref_* vary at the subsequent operating instants. The enlarged views show that *w* is adjusted in response to the parameter perturbations, *V_in_*, and the steps of *V_ref_*. Although the waveform becomes denser under these disturbances, *w* is still constrained within the admissible switching range, indicating that the proposed INTSMC law remains compatible with PWM-based implementation.

Figure 17d shows the response of the sliding variable sss. When *C*, *L*, *R*, *d*_1_(*t*), and *d*_2_(*t*) are changed at *t* = 0.2 s, the disturbed trajectory of *s* exhibits larger local oscillations around the sliding surface. Similar deviations can also be observed when the *V_in_* changes at *t* = 0.4 s and *t* = 0.6 s. For the *V_ref_* steps, *s* is temporarily driven away from zero, but it quickly returns to a small neighborhood of the sliding surface. Compared with the disturbance-free case, the disturbed case has stronger local oscillations because sss is affected by the combined effects of sensor-driver dynamics, *C*, *L*, *R*, *d*_1_(*t*), and *d*_2_(*t*). Even so, *s* remains bounded and convergent, confirming the robust reaching performance of the proposed controller under multi-source disturbances.

### 5.5. Comparative Evaluation of Different Sliding Mode Control Strategies Under Multi-Source Disturbances

**Figure 18:**
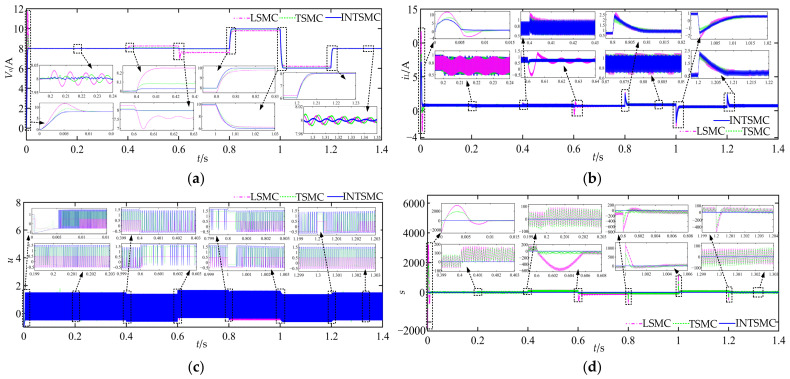
Comparison of different SMC methods considering sensor-driver dynamics and multi-source disturbances: (**a**) output voltage *V*_o_; (**b**) inductor current *i*_L_; (**c**) control law *w*; (**d**) sliding variable *s*.

To further verify the superiority of the proposed INTSMC, a comparative study is carried out among linear sliding mode control (LSMC), TSMC, and the proposed INTSMC. The three controllers are tested under the same multi-source disturbance and sensor-driver dynamic conditions. The corresponding responses of the output voltage, inductor current, control law, and sliding variable are shown in Figure 18a–d.

Figure 18a compares the output voltage responses. During the initial startup process, LSMC shows a larger overshoot and a slower recovery tendency, while TSMC improves the transient response to some extent. In contrast, INTSMC reaches the reference value with smaller overshoot and smoother convergence. When parameter perturbations, input-voltage changes, and reference-voltage steps are introduced, the proposed INTSMC tracks the desired voltage more closely, especially around *t* = 0.4 s, *t* = 0.6 s, *t* = 0.8 s, *t* = 1.0 s, and *t* = 1.2 s. The enlarged views further show that INTSMC produces smaller voltage deviation and weaker steady-state oscillation than LSMC and TSMC.

Figure 18b gives the inductor current responses. Compared with LSMC and TSMC, INTSMC has a smaller initial current overshoot and a faster damping process after the startup stage. Under multi-source disturbances, all three controllers generate current variations to rebalance the converter power flow. However, the current under LSMC exhibits larger oscillations in several enlarged regions, while TSMC still shows noticeable transient fluctuation. The proposed INTSMC maintains a more compact current response and suppresses the disturbance-induced current ripple more effectively.

The control laws of the three methods are shown in Figure 18c. LSMC and TSMC present more irregular switching behavior around disturbance and reference transition instants, especially when the operating point changes. In comparison, the control signal generated by INTSMC remains more continuous in the enlarged local regions and can adjust rapidly according to the variation of *V_in_*, *V_ref_*, and circuit parameters. Although the INTSMC control law contains high-frequency switching caused by PWM realization, its overall adjustment is more stable and better matched with the required converter response.

Figure 18d compares the sliding variable *s*. The LSMC and TSMC cases exhibit larger deviations from the sliding surface during startup and under sudden changes of *V_in_* and *V_ref_*. In particular, LSMC produces stronger oscillations and slower attenuation after some operating transitions. By contrast, the sliding variable under INTSMC remains closer to zero in most intervals and returns to the sliding neighborhood more rapidly after disturbance injection or reference variation. These results indicate that the proposed INTSMC has better reaching performance and stronger robustness than LSMC and TSMC under the combined influence of multi-source disturbances and sensor-driver dynamics.

### 5.6. Phase-Trajectory-Based Convergence-Time Evaluation

To further verify the response-time estimation method based on PTs, several representative initial points are selected according to the possible trajectory distribution shown in Figure 10. These initial points are located in different regions separated by the INTSMC sliding manifold, the critical surfaces *f_CS_*_1_ and *f_CS_*_2_, and the coordinate axes. For each initial point, both the reference PTs and the real PTs are plotted in two-dimensional and three-dimensional forms, where *x*_1_^∗^, *x*_2_^∗^, and *x*_3_^∗^ are used to describe the trajectory evolution under sensor-driver dynamics. The comparison is used to examine whether the real trajectory follows the predicted reaching direction and whether the proposed estimation formula can give a reasonable approximation of the actual convergence time.

**Figure 19:**
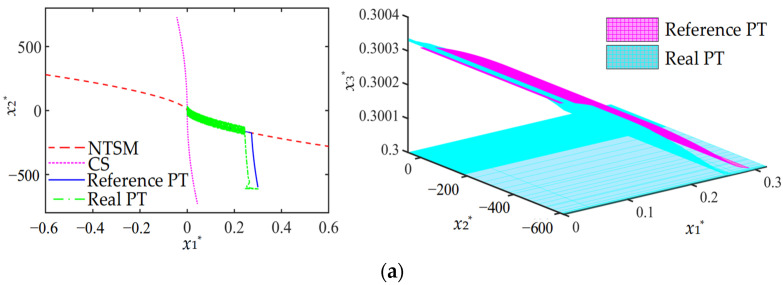

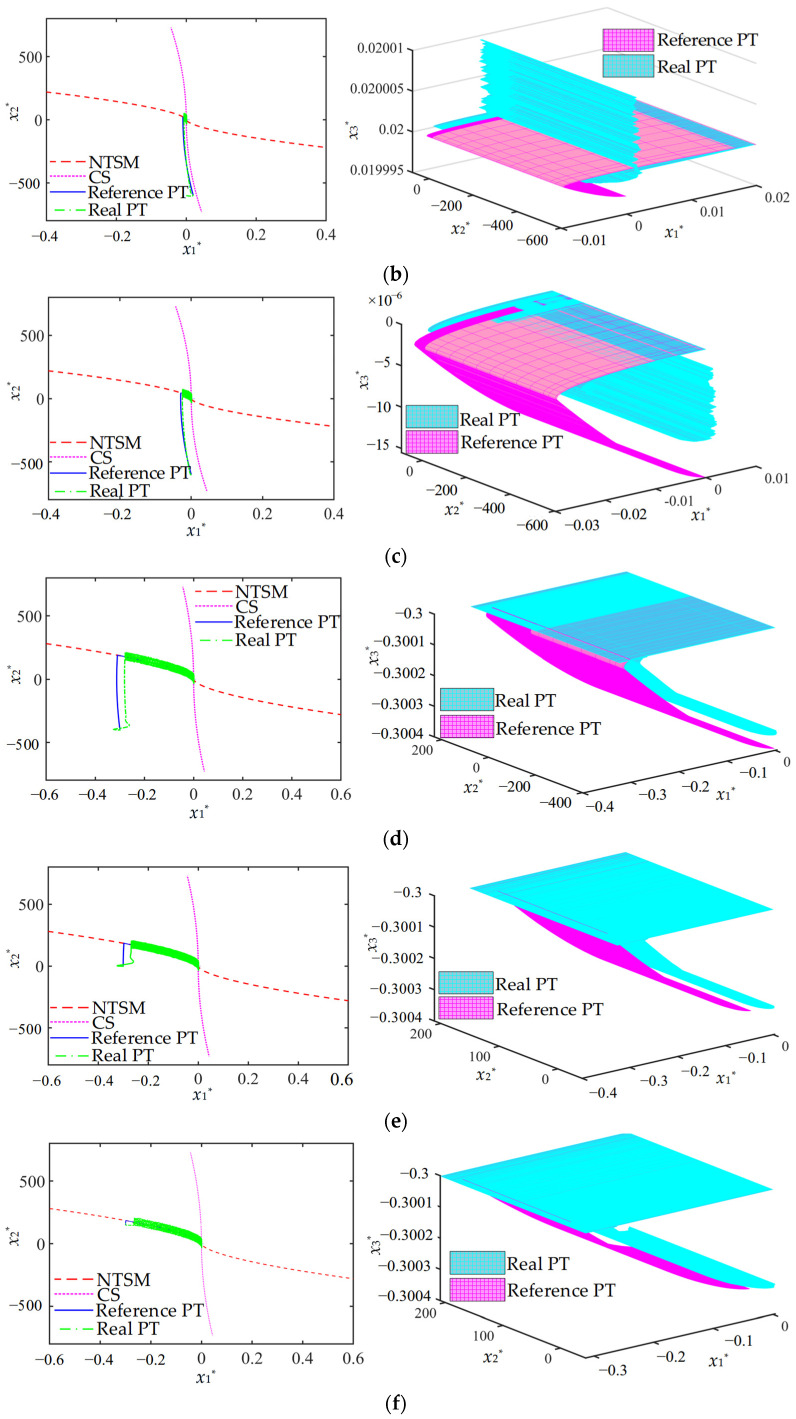

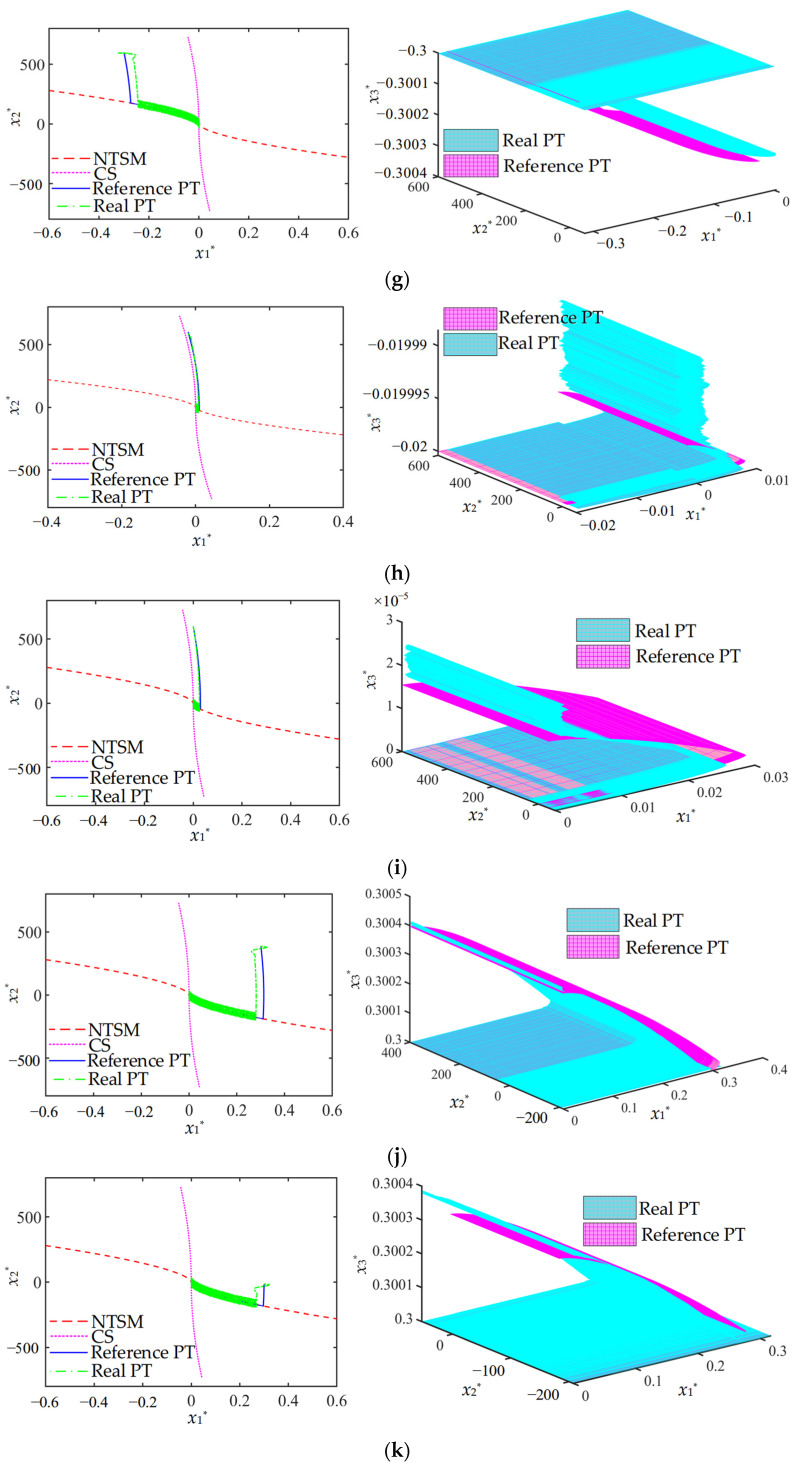

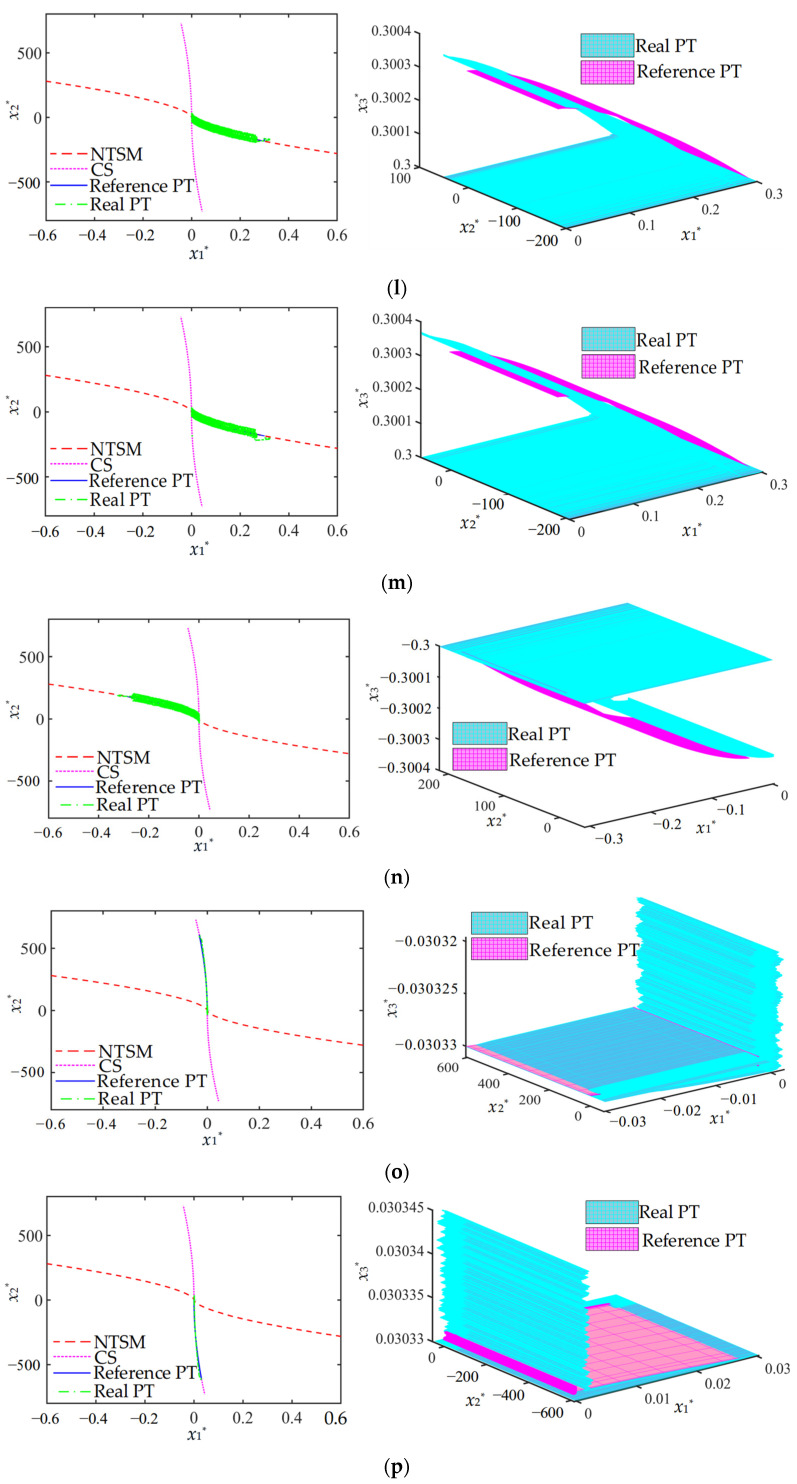
PTs from representative initial points: (**a**) A (0.3, −600); (**b**) B (0.02, −600); (**c**) C (0, −600); (**d**) D (−0.3, −400); (**e**) E (−0.3, 0); (**f**) F (−0.3, 150); (**g**) G (−0.3, 600); (**h**) H (−0.02, 600); (**i**) I (0, 600); (**j**) J (0.3, 400); (**k**) K (0.3, 0); (**l**) L (0.3, −150); (**m**) M (0.3, −184.9); (**n**) N (−0.3, −184.9); (**o**) Q (−0.03, 607.6); (**p**) R (0.03, −607.6).

As shown in Figure 19, the initial points in different regions follow different approaching paths, but all trajectories finally move toward the nonsingular terminal sliding manifold and converge to the origin or its small neighborhood. For the points in the right-half plane, such as A, B, J, K, L, M, and R, the trajectories first evolve according to the initial sign of *s*, and then bend toward the sliding manifold. For the points in the left-half plane, such as D, F, G, N, and Q, the trajectories are guided into the admissible reaching region before approaching the sliding surface. When the initial point is close to the critical surfaces *f_CS_*_1_ or *f_CS_*_2_, the trajectory does not directly cross the corresponding CS surface but evolves along the allowable side and then turns toward the sliding manifold, which agrees with the trajectory classification in Figure 10.

The 3D plots further show that the real PTs are close to the reference ones, with only small deviations caused by sensor-driver dynamics and switching ripple. The convergence-time comparison in Table 4 also shows that the estimated times are close to the simulated ones, supporting the effectiveness of the proposed PT-based estimation method in (61) and (74).

The relative estimation errors in Table 4 are all within 5%, indicating that the proposed PT-based estimation method can provide a close prediction of the convergence time for different initial regions. This result also suggests that the method is not limited to a single initial condition, but can characterize the reaching behavior from different phase-plane regions. This is particularly useful for micro-actuator-oriented power interfaces, where the allowable voltage recovery time is usually limited by the response margin of the actuator-side driving and control circuits.

## 6. Experimental Verification

**Figure 20:**
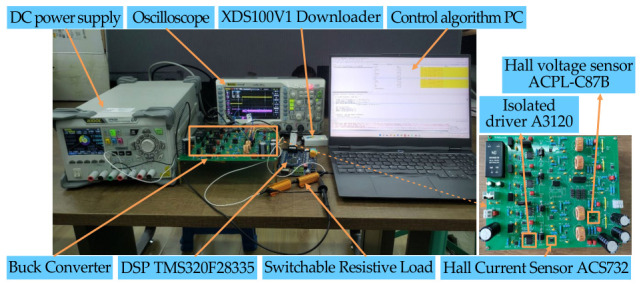
DSP-based buck converter HIL experimental platform.

To further verify the practical applicability of the sensor-driver-aware INTSMC scheme, a DSP TMS320F28335-based hardware-in-the-loop (HIL) experimental platform is established, as shown in Figure 20. The platform consists of a DC power supply, buck converter board, DSP controller, XDS100V1 downloader, control-algorithm PC, oscilloscope, and switchable resistive load. As labeled in Figure 20, the buck converter board uses the Hall voltage sensor ACPL-C87B, Hall current sensor ACS732, and isolated driver A3120, which are consistent with the device types adopted in the preceding sensor-driver dynamic modeling and identification analysis. The controllers are implemented in the DSP through fixed-frequency PWM, with both the PWM switching frequency and control sampling frequency set to 10 kHz. The main converter circuit parameters and controller parameters are kept consistent with those used in the simulation section. Based on this platform, four experimental tests are conducted, including input-voltage variation, load-resistance variation, reference-voltage variation, and identified sensor-driver dynamic variation. The first three tests compare LSMC, TSMC, and the sensor-driver-aware INTSMC scheme, while the fourth test evaluates the influence of sensor-driver dynamic variations under the INTSMC control framework.

### 6.1. Experimental Comparison Under Input-Voltage V_in_ Variations

**Figure 21:**
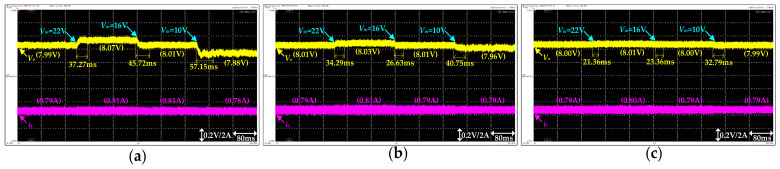
Output performance of different control strategies under *V_in_* variations: (**a**) LSMC; (**b**) TSMC; (**c**) INTSMC.

To evaluate the source-side disturbance rejection capability of different control strategies, the input-voltage variation experiment is first carried out on the DSP-based hardware platform. In this test, the output voltage reference is kept at *V_ref_* = 8 V, and the load resistance is fixed at *R_L_* = 10 Ω. The input voltage is changed sequentially as *V_in_*: 16 V → 22 V → 16 V → 10 V, where the low-input condition *V_in_* = 10 V is used to further examine the duty-ratio regulation capability under a reduced voltage margin. Figure 21 shows the measured output voltage *V_o_* and inductor current *i_L_* under LSMC, TSMC, and the sensor-driver-aware INTSMC scheme.

As shown in Figure 21a, LSMC can maintain voltage regulation after input-voltage changes, but it exhibits relatively long recovery processes and larger steady-state deviations. The measured settling times under the three input-voltage transitions are 37.27 ms, 45.72 ms, and 57.15 ms, respectively. The corresponding steady-state output-voltage values are 8.07 V, 8.01 V, and 7.88 V, giving a maximum steady-state error of 0.12 V. For TSMC in Figure 21b, the settling times are reduced to 34.29 ms, 26.63 ms, and 40.75 ms, and the maximum steady-state error is reduced to 0.04 V. In contrast, the sensor-driver-aware INTSMC in Figure 21c achieves faster recovery and smaller voltage deviation under all input-voltage transitions. Its measured settling times are 21.36 ms, 23.36 ms, and 32.79 ms, while the steady-state output voltage remains close to 8 V, with a maximum steady-state error of only 0.01 V. Compared with LSMC, the average settling time is reduced from 46.71 ms to 25.84 ms, corresponding to an improvement of about 44.7%. Compared with TSMC, the average settling time is reduced by about 23.8%. Meanwhile, the maximum steady-state voltage error is reduced by about 91.7% compared with LSMC and 75.0% compared with TSMC. The inductor current remains around 0.79 A~0.81 A in all three tests, indicating that the proposed scheme improves voltage recovery without introducing obvious current excursion. These results demonstrate that the sensor-driver-aware INTSMC has stronger input-disturbance rejection capability and better steady-state regulation performance under source-side voltage variations.

### 6.2. Experimental Comparison Under Load-Resistance R_L_ Variations

**Figure 22:**
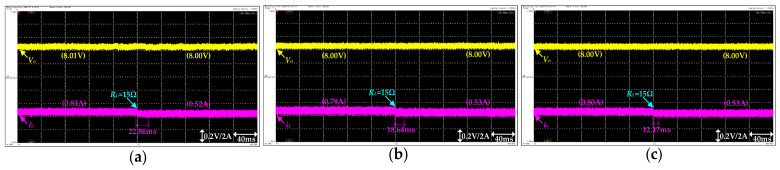
Output performance of different control strategies under *R_L_* variations: (**a**) LSMC; (**b**) TSMC; (**c**) INTSMC.

To verify the load-side disturbance rejection capability, the load-resistance variation experiment is carried out under *V_in_* = 16 V and *V_ref_* = 8 V. During the test, the load resistance is changed from *R_L_* = 10 Ω to *R_L_* = 15 Ω. The corresponding theoretical load current changes from approximately 0.8 A to 0.53 A. Figure 22 shows the experimental output voltage *V_o_* and inductor current *i_L_* responses under LSMC, TSMC, and the sensor-driver-aware INTSMC scheme.

As shown in Figure 22, all three control strategies can restore the output voltage to the reference value after the load resistance changes. However, their transient recovery speeds are different. Under LSMC, the measured settling time is 22.86 ms, and the output voltage changes from 8.01 V to 8.00 V, showing a small steady-state error within 0.01 V. For TSMC, the settling time is reduced to 18.64 ms, while the output voltage remains around 8.00 V. In comparison, the sensor-driver-aware INTSMC achieves the fastest recovery, with a settling time of 12.17 ms, and the output voltage is maintained at approximately 8.00 V before and after the load change. Compared with LSMC and TSMC, the settling time is reduced by about 46.8% and 34.7%, respectively. Meanwhile, the measured inductor current decreases from about 0.80 A to 0.53 A, which is consistent with the expected current variation caused by the load-resistance increase. These results indicate that the sensor-driver-aware INTSMC can maintain accurate voltage regulation and faster transient recovery under load-side disturbances.

### 6.3. Experimental Comparison Under Reference-Voltage V_ref_ Variations

**Figure 23:**
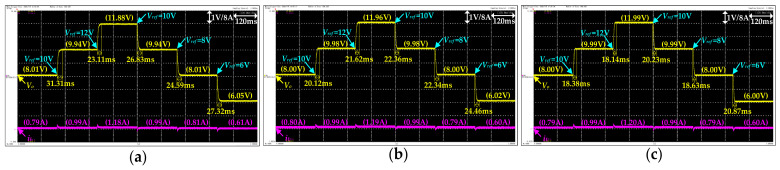
Output performance of different control strategies under *V_ref_* variations: (**a**) LSMC; (**b**) TSMC; (**c**) INTSMC.

To further verify the reference tracking capability of different controllers, the reference-voltage variation experiment is conducted under *V_in_* = 16 V and *R_L_* = 10 Ω. In this test, the output-voltage reference is changed successively as *V_ref_*: 8 V → 10 V → 6 V → 8 V. According to the voltage-divider coefficient *β* = 0.25, the corresponding controller reference voltage changes as *V_ref_*: 2.0 V → 2.5 V → 1.5 V → 2.0 V. The measured output voltage *V_o_* and inductor current *i_L_* under LSMC, TSMC, and the sensor-driver-aware INTSMC scheme are shown in Figure 23.

As shown in Figure 23, all three control strategies can track the changed reference voltage, but their transient response characteristics are different. LSMC shows relatively slow recovery and more obvious voltage deviation after each reference step, while TSMC improves the convergence speed compared with LSMC. In contrast, the sensor-driver-aware INTSMC achieves faster reference tracking and smaller steady-state deviation in all five *V_ref_* transitions. The measured settling times of INTSMC are 18.38 ms, 18.14 ms, 20.23 ms, 18.63 ms, and 20.87 ms, respectively. Meanwhile, the steady-state voltage error is maintained within 0.01 V, and the inductor current changes consistently with the output power demand without obvious excessive current excursion. Compared with LSMC and TSMC, the average settling time is reduced from 26.63 ms and 22.18 ms to 19.25 ms, corresponding to reductions of about 27.72% and 13.21%, respectively. The maximum steady-state voltage error is also reduced from 0.12 V for LSMC and 0.04 V for TSMC to 0.01 V for INTSMC. These results indicate that the sensor-driver-aware INTSMC has better transient tracking ability and steady-state regulation accuracy under continuous reference-voltage variations.

To further verify the PT-based response-time estimation method, the theoretical response time calculated by (73) and (74) is compared with the experimentally measured settling time of the sensor-driver-aware INTSMC. The comparison results are listed in Table 5. Since the experimental platform contains sampling delay, sensor noise, driver delay, component parasitics, and oscilloscope reading uncertainty, the experimental error is slightly larger than that in the simulation comparison. Nevertheless, the relative error remains within an acceptable range, indicating that the estimation method in (73) and (74) can provide a useful prediction of the practical convergence process.

As shown in Table 5, the estimated response times obtained from (73) and (74) are slightly lower than the experimentally measured settling times. The relative errors are 11.39%, 10.81%, 13.21%, 12.50%, and 13.98%, respectively, with a maximum value of 13.98%. This deviation is reasonable because the PT-based response-time estimation is derived from the simplified phase-trajectory analysis, whereas the hardware experiment contains sampling delay, sensor noise, driver delay, component parasitics, power-supply fluctuation, and oscilloscope reading uncertainty. Nevertheless, the estimated values remain close to the measured settling times and correctly reflect the convergence trend under continuous reference-voltage variations. Therefore, the comparison verifies that the response-time estimation method in (73) and (74) can provide a useful prediction of the practical convergence process of the sensor-driver-aware INTSMC.

**Figure 24:**
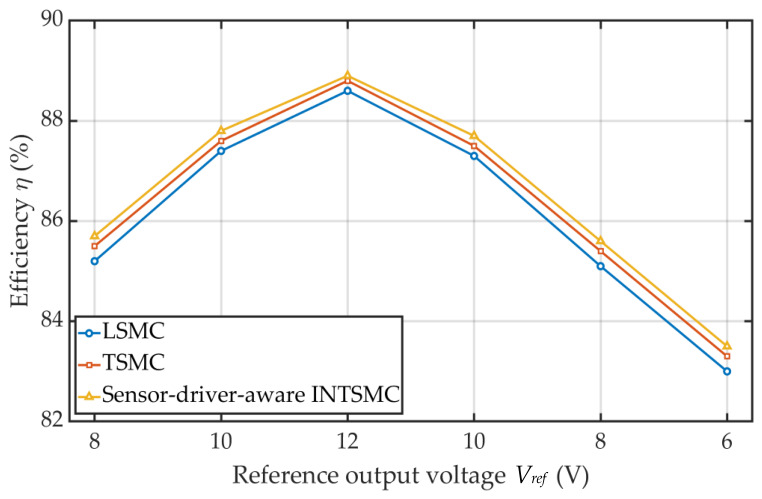
Experimental efficiency comparison under *V_ref_* variations.

To further evaluate whether the improved transient tracking performance is achieved at the cost of converter efficiency, the experimental efficiency is also calculated at each steady-state operating point under the reference-voltage variation test. The efficiency is obtained by *η* = *V_o_I_o_*/(*V_in_I_in_*) × 100%. The efficiency comparison of LSMC, TSMC, and the sensor-driver-aware INTSMC is shown in Figure 24.

As shown in Figure 24, the efficiencies of the three controllers show a similar trend under different reference-voltage levels. The efficiency increases as *V_ref_* rises from 6 V to 12 V, mainly because the output power increases and the proportion of fixed hardware losses decreases. For the sensor-driver-aware NTSMC, the measured efficiency remains within 83.5~88.9%. This range is close to those of LSMC (83.0~88.6%) and TSMC (83.3~88.8%). Therefore, the sensor-driver-aware INTSMC improves the transient response and steady-state tracking accuracy without introducing an obvious efficiency penalty.

### 6.4. Startup Verification Under Identified Sensor-Driver Dynamic Variations

**Figure 25:**
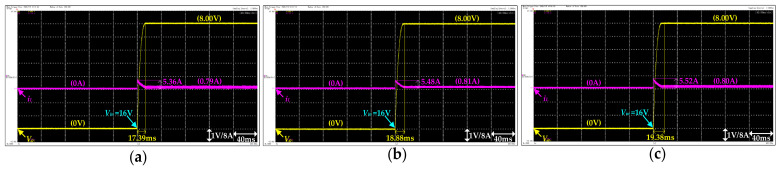
Output performance of different control strategies when *V_ref_* changes: (**a**) *μ_x_*_1_, *μ_x_*_2_, *μ_xu_*; (**b**) 4*μ_x_*_1_, 4*μ_x_*_2_, 4*μ_xu_*; (**c**) 8*μ_x_*_1_, 8*μ_x_*_2_, 8*μ_xu_*.

To further verify the influence of sensor-driver dynamics on the hardware response, a startup experiment is carried out under the sensor-driver-aware INTSMC scheme. In this test, the input voltage is switched from 0 V to 16 V, while the output-voltage reference is set as *V_ref_* = 8 V and the load resistance is kept at *R_L_* = 10 Ω. It should be emphasized that the hardware platform already contains the inherent dynamics of the Hall voltage/current sensors, analog-to-digital converter (ADC) sampling, PWM module, isolated driver, and power switch. To reproduce possible response-time variations of practical sensing and driving links in a repeatable and model-consistent way, the identified sensor-driver dynamic models in Table 2 are embedded in the HIL implementation. The static gains of the identified models are kept unchanged, while the response-time parameters *μ_x_*_1_, *μ_x_*_2_, and *μ_u_* are simultaneously adjusted. Three cases are tested, namely the nominal setting (*μ_x_*_1_, *μ_x_*_2_, *μ_u_*), the fourfold setting (4*μ_x_*_1_, 4*μ_x_*_2_, 4*μ_u_*), and the eightfold setting (8*μ_x_*_1_, 8*μ_x_*_2_, 8*μ_u_*). The corresponding startup responses are shown in Figure 25.

As shown in Figure 25a, under the nominal sensor-driver dynamic setting, the output voltage rises from 0 V to 8.00 V with a measured settling time of 17.39 ms, and the inductor-current peak during startup is about 5.36 A. When the response-time parameters are increased to 4*μ_x_*_1_, 4*μ_x_*_2_, and 4*μ_u_*, as shown in Figure 25b, the settling time increases slightly to 18.88 ms, and the startup current peak increases to about 5.48 A. Under the more conservative dynamic setting (8*μ_x_*_1_, 8*μ_x_*_2_, 8*μ_u_*), Figure 25c shows that the settling time further increases to 19.38 ms, while the current peak reaches about 5.52 A. Compared with the nominal setting, the settling time increases by about 8.57% and 11.44% under the fourfold and eightfold dynamic settings, respectively. Meanwhile, the steady-state output voltage is still maintained at approximately 8.00 V, and the steady-state current remains around 0.79 A~0.81 A. These results indicate that slower sensor-driver dynamics slightly delay the startup response and increase the transient current peak, but the sensor-driver-aware INTSMC can still ensure stable voltage establishment and accurate steady-state regulation. Therefore, the experiment confirms the practical robustness of the proposed sensor-driver-aware design under model-consistent sensor-driver dynamic variations.

## 7. Conclusions

This paper presented the analysis and design of a sensor-driver-aware INTSMC strategy for buck converter power interfaces in micro-actuator systems. A control-oriented model was first established by incorporating multi-source disturbances, circuit parameter perturbations, Hall sensor dynamics, and isolated driver dynamics, so that the non-ideal relationship among the real system states, measured feedback signals, and effective switching action could be described more explicitly. Based on this model, an integral nonsingular terminal sliding surface and a PWM-realizable control law were designed to improve voltage regulation performance while avoiding the singularity problem in TSMC dynamics. The stability and existence conditions were analyzed by considering both multi-source disturbances and sensor-driver-induced dynamic deviations. In addition, a PT-based response-time estimation method was introduced to evaluate the reaching behavior from different initial regions. Simulation results showed that the proposed INTSMC can maintain accurate voltage tracking, bounded current response, and robust sliding convergence under sensor-driver dynamics, parameter perturbations, input-voltage variations, reference-voltage changes, and time-varying disturbances. Compared with LSMC and TSMC, the proposed method achieved a smoother transient response, smaller voltage deviation, and stronger disturbance rejection. These results suggest that the proposed approach is a feasible control solution for local power regulation in compact micro-actuator-oriented microsystems.

## Figures and Tables

**Table 1:** Circuit parameters of the buck converter.

Description	Parameter	Value
Input-voltage variation range	*V_in_*	12~20 V
Inductor	*L*	1 mH
Capacitor	*C*	3200 μF
Load-resistance variation range	*R_L_*	10~12 Ω
Reference output voltage range	*V_ref_*/*β*	1.5~2.5 V
Output-voltage regulation range	*V_o_*	6~10 V
Divider resistor	*R*_1_	1 kΩ
Variable divider resistor	*R*_2_	[1 kΩ, 9 kΩ]
Variable resistor	*R_m_*	[1 kΩ, 9 kΩ]
PWM switching frequency	*f_s_*	10 kHz

**Table 2:** Identifying the model information of the sensor drivers.

Device Type	Identification Model	Rise Time (μs)	Static Linear Gain	Other Parameters
Hall current sensor ACS732	$y\;=\;\frac{1.186\;\times \;10^{11}}{s^{2}\;+\;1.575\;\times \;10^{5}s\;+\;1.173\;\times \;10^{11}}$	*μ_x_*_1_ = 2.9181	*h_x_*_1_ = 0.9902	*P_x_*_1_ = 1.0099 *Q_x_*_1_ = 0.4596
Hall voltage sensor ACPL-C87B	$y\;=\;\frac{3.949\;\times \;10^{11}}{s^{2}\;+\;5.505\;\times \;10^{5}s\;+\;3.913\;\times \;10^{11}}$	*μ_x_*_2_ = 1.5991	*h_x_*_2_ = 0.9903	*P_x_*_2_ = 1.0098 *Q_x_*_2_ = 0.8802
Isolated driver A3120	$y_{3}\;=\;\frac{1.128\;\times \;10^{11}}{s^{2}\;+\;5.956\;\times \;10^{5}s\;+\;1.117\;\times \;10^{11}}$	*μ_u_* = 2.9920	*h_u_* = 0.9903	*P_u_* = 1.0098 *Q_u_* = 1.7820

**Table 3:** Multi-source disturbance settings for the sensor-driver-aware buck converter.

Time	Category	Parameter Setting	Description
*t* < 0.2 s	Initial operating condition	*C* = 3.2 mF, *L* = 1 mH, *R* = 10 Ω, E = 16 V, *V_ref_* = 8 V, *d*_1_(*t*) = *d*_2_(*t*) = 0	The system operates under nominal conditions and reaches the initial steady state.
*t* = 0.2 s	Circuit parameter perturbations	*C*: 3.2 mF → 2.9 mF; *L*: 1 mH → 0.9 mH; *R*: 10 Ω → 11 Ω	The parameter perturbations are introduced simultaneously and maintained afterwards.
*t* = 0.2 s	Time-varying noise disturbances	Amplitudes of *d*_1_(*t*) and *d*_2_(*t*): 0 → 0.15	The time-varying disturbances are injected together with the parameter perturbations and maintained afterwards.
*t* = 0.4 s	Input-voltage variation	*V_in_*: 16 V → 20 V	The input voltage is increased to test source-side disturbance rejection.
*t* = 0.6 s	Input-voltage variation	*V_in_*: 20 V → 12 V	The input voltage is decreased to further test source-side robustness.
*t* = 0.8 s	Input-voltage recovery	*V_in_*: 12 V → 16 V	The input voltage returns to the nominal value.
*t* = 0.8 s	Reference-voltage change	*V_ref_*: 8 V → 10 V	The output voltage reference is increased.
*t* = 1.0 s	Reference-voltage change	*V_ref_*: 10 V → 6 V	The output voltage reference is decreased.
*t* = 1.2 s	Reference-voltage recovery	*V_ref_*: 6 V → 8 V	The output voltage reference returns to the nominal value.

**Table 4:** Comparison between simulated and estimated convergence times for different initial points.

Initial Point	Region Characteristic	*t_r_*/ms (Simulation)	*t_r_*/ms (Estimate)	Estimation Error ^1^/%
A (0.30, −600)	Right-lower region, near *f_CS_*_1_	3.42	3.55	3.80
B (0.02, −600)	Near the negative *x*_2_^∗^-axis	2.18	2.25	3.21
C (0, −600)	Negative *x*_2_^∗^-axis	2.06	2.13	3.40
D (−0.30, 300)	Left-lower region	3.05	2.92	4.26
E (−0.30, 0)	Negative *x*_1_^∗^-axis	2.64	2.75	4.17
F (−0.30, 150)	Left-upper region	2.88	3.01	4.51
G (−0.30, 600)	Far left-upper region	4.08	4.26	4.41
H (−0.02, 600)	Near the positive *x*_2_^∗^-axis	2.74	2.86	4.38
I (0, 600)	Positive *x*_2_^∗^-axis	2.52	2.62	3.97
J (0.30, 400)	Right-upper region	3.76	3.91	3.99
K (0.30, 0)	Positive *x*_1_^∗^-axis	2.34	2.45	4.70
L (0.30, −150)	Right-lower region	2.58	2.68	3.88
M (0.30, −184.9)	Right-lower region near *s* = 0	2.72	2.83	4.04
N (−0.30, −184.9)	Left-side region near *s* = 0	3.18	3.31	4.09
Q (−0.03, 607.6)	Upper region near *f_CS_*_2_	2.93	3.06	4.44
R (0.03, −607.6)	Lower region near *f_CS_*_1_	2.61	2.73	4.60

^1^ Estimation error = |*t_r_*(simulation) − *t_r_*(estimate)|/*t_r_*(estimate) × 100%.

**Table 5:** Comparison between theoretical response-time estimation and experimental settling time under *V_ref_* variations.

*V_ref_* Variation	*t_r_*/ms (Experimental)	*t_r_*/ms (Estimate)	Estimation Error/%
8 V → 10 V	18.38	16.55	11.06
10 V → 12 V	18.14	16.37	10.81
12 V → 10 V	20.23	17.87	13.21
10 V → 8 V	18.63	16.56	12.50
8 V → 6 V	20.87	18.31	13.98

## Data Availability

Data are contained within the article.
